# Self-Healing Silsesquioxane-Based Materials

**DOI:** 10.3390/polym14091869

**Published:** 2022-05-02

**Authors:** Maria Nowacka, Anna Kowalewska

**Affiliations:** Centre of Molecular and Macromolecular Studies, Polish Academy of Sciences, Sienkiewicza 112, 90-363 Łódź, Poland; mnowacka@cbmm.lodz.pl

**Keywords:** polyhedral silsesquioxanes, POSS, self-healing, hybrid materials

## Abstract

This review is devoted to self-healing materials (SHM) containing polyhedral oligomeric silsesquioxanes (POSS) as building blocks. The synthetic approach can vary depending on the role POSS are expected to play in a given system. POSS (especially double-decker silsesquioxanes) can be grafted in side chains of a polymer backbone or used as segments of the main chain. Appropriate functionalization allows the formation of dynamic bonds with POSS molecules and makes them an active component of SHM, both as crosslinking agents and as factors that enhance the dynamics of macromolecules in the polymer matrix. The latter effect can be achieved by reversible release of bulky POSS cages or by the formation of separated inclusions in the polymer matrix through hydrophobic interactions and POSS aggregation. The unique properties of POSS-based self-healing systems make them interesting and versatile materials for various applications (e.g., repairable coatings, sealants, sensors, soft materials for tissue engineering, drug delivery, and wound healing).

## 1. Introduction

Polymeric self-healing materials (SHM) are of great interest due to their dynamic properties that can stabilize their performance extending the service life and thus reduce the impact of synthetic materials on the environment. SHM can self-heal spontaneously or stimulated by environmental conditions, after a fracture or external mechanical damage [1,2]. The long-term stability, self-healing ability, and flexibility of polymeric SHM make them valuable next-generation composite materials that can be used in a wide variety of environments and for applications as paints and coatings, sensors, actuators, as well as materials for electronics, healthcare (drug delivery, adhesives, tissue engineering, and wound healing), textiles, soft robotics, automotive, and aerospace [3,4]. The self-healing in reversible systems usually involves associative or dissociative exchange reactions with formation of secondary bonds (hydrogen [5] or halogen bonding [6], dynamic covalent transitions [7,8,9], π–π stacking [10], host-guest interactions [11,12], ion–dipole interactions [13], ionic aggregation [14], metal–ligand interactions [15,16], and hydrophobic interactions [17]) or chemical reactions (Diels–Alder reaction [18,19,20,21,22], Alder-ene reaction [23], disulfide metathesis [24,25,26,27], imine transamination [28,29], transesterification [30], boroxine [31], or acylhydrazones bonding [32], radical-based exchange systems [33] and electrochemical reactions [34]) that may occur with or without an external stimulus. These phenomena can reform the microstructure of a polymer matrix through multiple healing cycles. Nevertheless, intimate contact between the crack surfaces is required to allow sufficient time for the diffusion and interaction of components. Thus, the intrinsic ability of a polymer to self-heal may depend not only on the kinetics of dynamic bond exchange and interactions, but also on the mobility of macromolecular chains that may enable or impede structural reformation. In addition to the internal phenomena occurring in polymers and composites, SHM can also respond to external self-healing mechanisms. Such a self-repair process is considered irreversible and involves the introduction of healing agents embedded or carried in microcapsules or microvascular fibers. Once released through carrier rupture, the healing agents can react to bind the damaged parts and surfaces. However, deposition of foreign materials in a polymer matrix may present further technical challenges (a necessity for additional functionalization to improve dispersion of the additives, engineering of interface phenomena, localized/uneven healing and processing problems, degradation of mechanical and functional properties). Surface functionalization of nanoparticles directs interfacial phenomena, which can aid in the self-healing of polymer composites. The efficiency of this process can also be increased, apart from the healing conditions, by combining multiple mechanisms, e.g., shape memory-assisted self-healing, soft-hard microphase separation, or the addition of nanofillers. The latter can further increase the mechanical strength of the polymer composite and also initiate the healing process, e.g., by multi-complexation between the components [35].

**Scheme 1:**
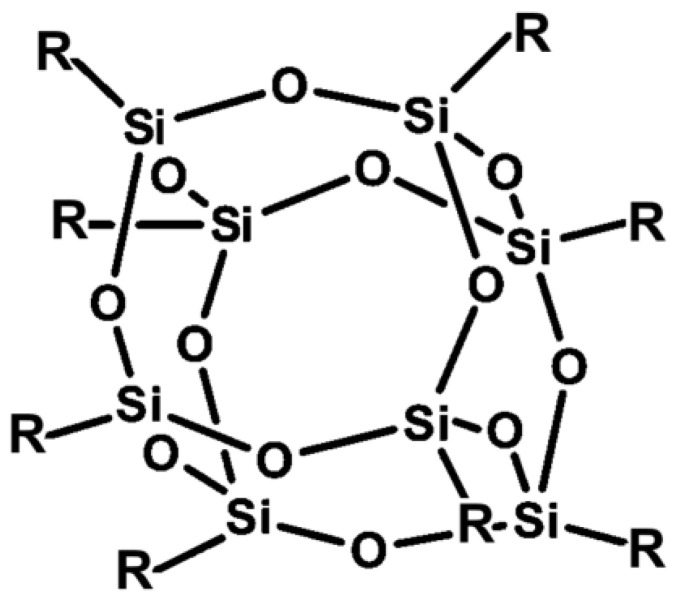
Schematic structure of a POSS molecule (R = H or an organic or heterorganic group).

**Scheme 2:**
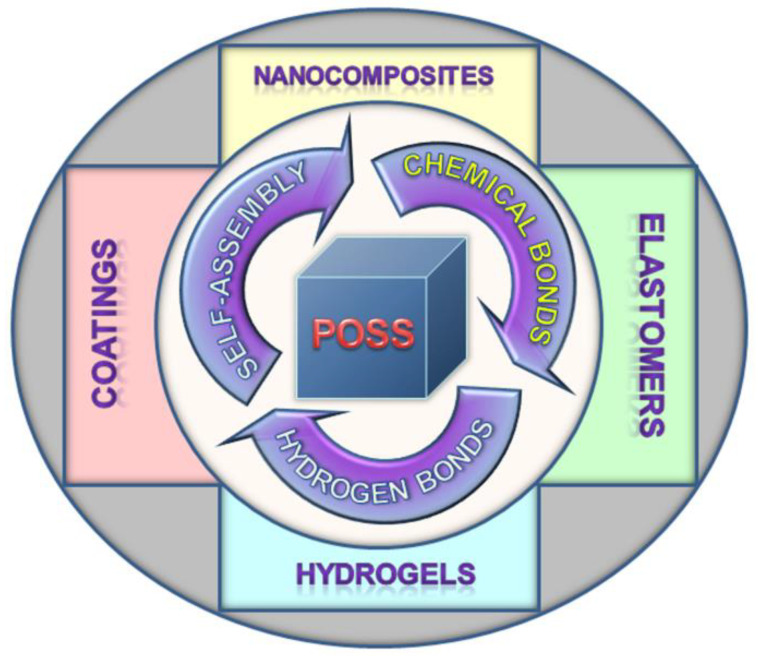
POSS-based SHM—self-healing phenomena and applications.

Polyhedral oligosilsesquioxanes (POSS) are the smallest known silica-like hybrid particles (1–3 nm diameter) with a well-defined inorganic framework made of silicon and oxygen atoms, and the empirical formula (RSiO_1.5_)_n_, where *n* = 6, 8, 12; R is a hydrogen atom or an organic or heterorganic group (Scheme 1) [36]. Their key features arise from the unique hybrid cage structures with a nanoscale inorganic core of uniform size and surrounded by eight functional groups. POSS have gained huge interest due to the fact that they can be functionalized quite easily to obtain materials with tunable structures and properties [37]. Their molecular self-assembly to form well-organized nanostructures is well known, and considerable efforts have been directed to the construction of POSS-based hybrid materials with precise architectures [38,39,40,41]. POSS are of great importance as nano building blocks in materials chemistry and engineering (including self-healing hybrid materials), optoelectronics, medicine, and catalysis [42]. In recent years, monodisperse POSS have also been widely used as components of hybrid polymer nanocomposites with enhanced thermal stability, hydrophobicity, mechanical properties, reduced flammability, and high resistance to oxygen plasma etching [43,44,45,46,47,48,49]. The number of papers devoted to the application of POSS in self-healing systems is steadily increasing. This review summarizes the ideas and research work carried out over the past decade in the synthesis and applications of self-healing hybrid materials containing polyhedral silsesquioxanes. This review is focused on the self-healing of POSS-containing materials (Scheme 2) that operate by phase separation and self-aggregation of silsesquioxane moieties as well as changes in mobility of macromolecules in the polymer matrix regulated by the dynamic exchange of bonds to POSS moieties. These effects can be further creatively exploited for the design of new-generations of smart materials including drug delivery systems, materials for tissue engineering, and hydrogels.

## 2. POSS-Based Self-Healing Nanocomposites

### 2.1. POSS Used as Specialty Additives

**Figure 1:**
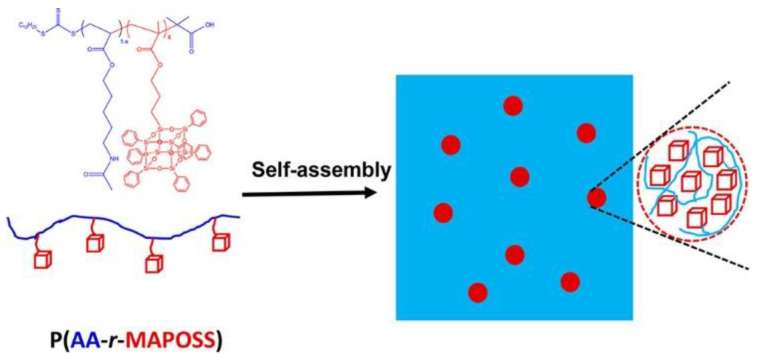
Phase separation on self-assembly of POSS covalently grafted to polymer chains. Reprinted with permission from *ACS Appl. Polym. Mater.* 2019, *1*, 359–368 [50]. Copyright (2019) American Chemical Society.

Polyhedral oligomeric silsesquioxanes can be used as additives to alter the properties of self-healing materials, although not directly affecting the dynamic process. POSS can be grafted into the side chains of macromolecules or can be added as fillers to the polymer matrix. The third approach is a combination of the above two methods. For example, hybrid nanocomposite elastomers with random [50] or block [51] structures were prepared by copolymerization of 5-acetylaminopentyl acrylate (AA) with 3-methacryloxypropylheptaphenyl polyhedral oligomeric silsesquioxane (MAPOSS). The presence of POSS resulted in enhancement of glass transition temperature (T_g_) and improvement of mechanical strengths. The composites were microphase-separated due to the aggregation of POSS units into nanodomains, which become the reinforcing net points of the physically crosslinked network (Figure 1). The POSS microdomains restricted the segmental movement of polyacrylate (PAA) chains which slowed down hydrogen bond exchange. Yet, the elastomeric nature of PAA was preserved despite the microphase separation. This particular structure was critical for the simultaneous shape memory (physical crosslinking) and self-healing properties (dynamic exchange of the intramolecular hydrogen bonds among amide groups of acrylate). As expected, the self-repair efficiency decreased slightly with increasing mass fraction of MAPOSS for both block and random copolymers.

Monofunctional epoxy-POSS and octaphenyl-POSS were used to reinforce the epoxy-amine network by producing nanocomposites with chemically bound pendant silsesquioxane side groups or physically doped POSS nanofillers [52]. The synergistic combination of these two types of POSS, one of which was reactive and the other non-reactive to the epoxy-resin, resulted in an effective reinforcement of the epoxy network through strong physical crosslinking due to polymer-POSS and POSS–POSS interactions. The epoxy resin/POSS nanocomposite was modified by introducing a maleimide-based thermoreversible Diels–Alder type material to produce a self-repairing interpenetrating network. The Diels-Alder (DA) “click” reaction involves a [4 + 2] cycloaddition between an electron-rich diene (e.g., furan, cyclopentane) and an electron-poor dienophile (e.g., maleimide, methacrylate) to form a stable product. The addition is thermally reversible [retro-Diels-Alder reaction (rDA)], and the dynamics of DA adducts can be easily controlled by temperature. The resulting reinforced nanocomposite (modulus of 800 MPa at room temperature) healed with an efficiency of 61%. The self-healing efficiency (EH) decreased with increasing POSS nanofiller content and heterogeneity of the hybrid material.

The doping of silsesquioxane additives into a polymeric material appears to be the simplest way to obtain nanocomposites. However, this approach requires appropriate functionalization of inorganic structures to ensure optimal miscibility and dispersion, as well as engineering of physical or chemical crosslinking. For example, POSS functionalized with amino or acid groups, capable of interactions based on labile ionic bond formation in methylvinylsilicone (MVQ) elastomer, were introduced into a rubber matrix filled with fumed silica [53]. Amic acid-isobutyl POSS (AAIb-POSS), aminopropylisobutyl-POSS (APIb-POSS), and aminoethylaminopropylisobutyl-POSS (AEAPIb-POSS) were used as additives to obtain self-healing effect through the formation of the thermoreversible network. The most significant improvement was observed for the system containing AAIb-POSS/APIb-POSS. The obtained composites had good mechanical properties and their conditioning at elevated temperature (70 °C for 24 h) appeared to have a significant effect on the self-healing properties. Gas permeability measurements showed that regeneration of composites formed with POSS after the conditioning favorably influenced their barrier properties. The observed reduction in gas permeability was attributed to an increase in the concentration of ionic groups induced by elevated temperature, which led to an increase in the density of crosslinks and consequently inhibited air permeation through the cured rubber composites.

**Figure 2:**
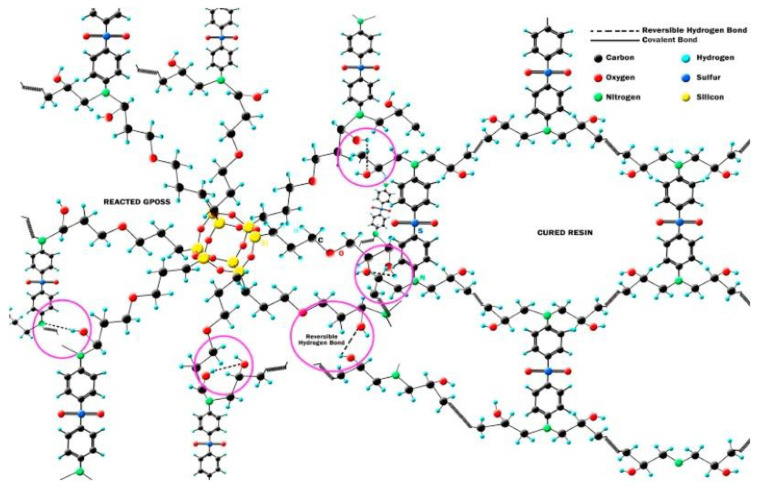
Self-healing mechanisms based on reversible hydrogen bonds in a phase separated epoxy resin containing POSS and MWCNT. Reprinted from *Compos. Part B* 2017, *128;* Guadagno, L.; Naddeo, C.; Raimondo, M.; Barra, G.; Vertuccio, L.; Sorrentino, A.; Binder, W.H.; Kadlec, M., Development of self-healing multifunctional materials, 30–38. Ref. [54] Copyright (2017), with permission from Elsevier.

Reversible hydrogen bonds resulting from interactions between the epoxy resin and POSS additives that were functionalized with different numbers of oxirane rings [octa(glycidyl)-POSS (GPOSS), octa(epoxycyclohexyl)-POSS (ECPOSS) and open cage tri(glycidyl)-penta(cyclohexyl)-POSS (TCPOSS)] as well as dodecaphenyl-POSS (DPHPOSS), were used to activate self-healing mechanisms in a thermosetting multifunctional resin containing embedded multi-walled carbon nanotubes (MWCNT) [54]. GPOSS nanofillers reduce the viscosity of the epoxy resin matrix, counterbalancing the viscosity increase caused by MWCNT. Proper design of the phase composition with hybrid POSS (Figure 2) allowed an increase in EH of up to 400%. As shown by the results of dynamic mechanical analysis (DMA), MWCNT induced chain mobility in domains where reversible hydrogen bonds between the epoxy resin and POSS provided enhanced healing efficiency.

It should be noted that in some cases the phase separation caused by the propensity of POSS to aggregate can be creatively exploited to alter unwanted effects caused by excessive supramolecular interactions. An example can be the alternating supramolecular thermoplastic poly(lactide)/poly(ε-caprolactone) triblock copolymers admixed with 1,2-propanediol-heptaisobutyl-POSS. PLA-PCL-PLA were functionalized at their end positions with 2-ureido-4[1H]-pyrimidinone (UPy) groups, which are capable of forming self-complementary quadruple hydrogen bonds [55]. The use of strongly associative quadruple hydrogen bonds allows not only to improve the thermomechanical properties of polymers, but also to engineer their intrinsic self-healing properties, which is of great interest. Such quadruple associates in supramolecular copolymers can undergo intensive dynamic and reversible exchange, which can be exploited for self-regeneration [56,57,58,59]. In this case, the incorporation of UPy units hindered the crystallization of polymer chains, while the addition of POSS molecules weakened the effect of the inhibition process by inducing heterogeneous nucleation.

**Figure 3:**
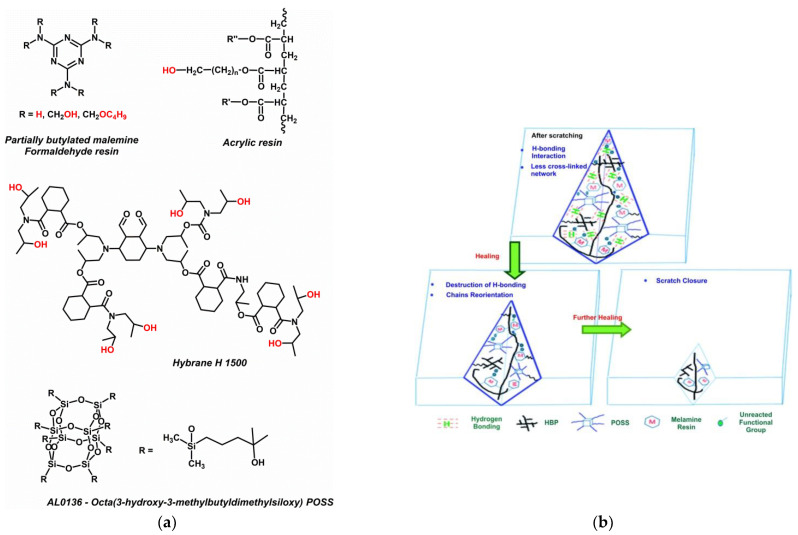
(**a**) Chemical structure of acrylic, melamine, HBP and POSS components used in clearcoat formulations; (**b**) Schematic illustration of enhanced healing in presence of HBP and POSS. Reproduced from *RSC Adv*. 2016, *6*, 76028–76041 [60] with permission from the Royal Society of Chemistry.

It has been also shown that the scratch resistance and healing ability of an acrylic melamine clearcoat can be improved upon the addition of hyperbranched polymer (HBP) and POSS bearing eight (3-hydroxy 3-methyl butyl) groups (Figure 3) [60]. The observed effect is based on simultaneous reinforcement with covalent and physical bonds. The enhanced scratch resistance was attributed to the increased elastic recovery of the clearcoats due to the introduction of POSS, while their self-healing properties were attributed to the partial replacement of covalent crosslinks with hydrogen bonds in the presence of modifiers. The control of chemical and physical interactions in the network was enabled by the addition of different loadings of modifiers. The resulting more flexible crosslinked network maintained its mechanical integrity at ambient temperature, but the chain motions of the macromolecular components were dynamic enough to allow healing at high temperatures.

### 2.2. Hydrophobic Coatings

Liquid-repellent surfaces (water contact angles >150° and low roll-off angles; both superhydrophobic and superoleophobic), which are able to perform non-stick, self-cleaning, and anti-pollution functions, have recently attracted much attention in materials engineering. Unfortunately, most super-repellent surfaces exhibit poor durability after exposure to chemical environments (including air oxidation), UV radiation, or physical damage (mechanical abrasion). It would be a significant advantage if synthetic coatings could recover their intrinsic hydrophobicity without modification or the addition of low surface energy substances. Recently, significant progress has been made in developing robust, self-healing superhydrophobic surfaces and fabrics with enhanced durability. Functionalized polyhedral oligosilsesquioxanes can help maintain these special properties of self-repairing coatings [61]. The self-healing process of this type is based on the recovery of low-surface energy and/or topological structures.

The incorporation of highly perfluorinated compounds into the coating allows for achieving low surface energy without the need for specific chemical surface grafting. It was found that superhydrophobic and superoleophobic fabrics coated with tridecafluorooctyl triethoxysilane (FAS) hydrolysate with embedded but well-dispersed particles of octa(1H,1H,2H,2H-heptadecafluorodecyl)octa-silsesquioxane (FD–POSS) can self-regenerate during heating, showing excellent durability against physicochemical (acid exposure, UV light) and mechanical (machine wash, abrasion) factors [62]. The proposed self-healing mechanism is based on the enhanced mobility of FD-POSS molecules at elevated temperatures. As a result of molecular rotation and movement, the hydrophobic fluorinated alkyl chains become exposed to the surface, minimizing the surface free energy, while the more polar groups that appear after the chemical damage of the surface, tend to hide inside the coating layer. The movement of POSS was somewhat reduced by the surrounding hydrolyzed FAS resin, and this effect stabilized the surface properties.

FD-POSS and FAS were also doped into 3,4-ethylenedioxythiophene (EDOT), which was used to prepare a robust, electrically conductive, superamphiphobic fabric by vapor-phase polymerization [63]. The incorporation of FD-POSS and FAS into the PEDOT layer did not change its conductivity but significantly improved its stability to washing (500 cycles of standard laundry) and abrasion (10 000 cycles). The coated fabric had contact angles of 169° and 156°, respectively to water and hexadecane. Furthermore, the coating was shown to self-heal after chemical damage, restoring the liquid repellency. A superamphiphobic surface was also obtained by coating a fabric substrate with fluoroalkyl surface-modified silica nanoparticles (FS-NP), followed by the addition of a topcoat made from a mixture of FAS and FD-POSS [64]. A short thermal treatment (5 min at 140 °C) accelerated the transport of FAS to the damaged area to minimize the surface energy. Superamphiphobicity was restored by topological surface roughness reconstruction with FD-POSS and SiO_2_ nanoparticles that occurred after heating the abraded surface at 140 °C for 30 min. A similar effect was obtained for self-healing waterborne superhydrophobic coatings prepared from a polysiloxane emulsion containing fluorinated POSS and modified SiO_2_ nanoparticles [65].

**Figure 4:**
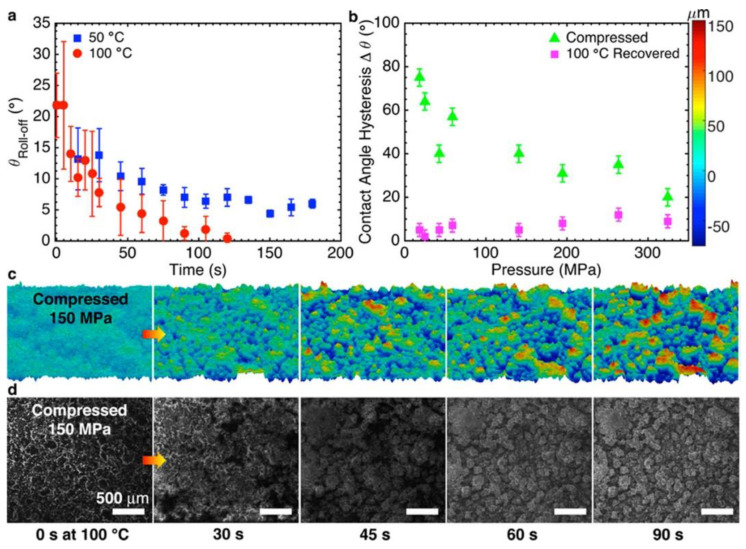
Texture recovery of FPU/FD-POSS coatings (**a**) self-healing as a function of time and temperature, after 1000 Taber abrasion cycles. (**b**) contact angle hysteresis for the coatings before and after thermal recovery from compression, as a function of the compressive load. (**c**) height maps of the coatings after 150 MPa compression, as a function of recovery time at 100 °C (**d**). Reprinted with permission from *ACS Appl. Mater. Interfaces* 2017, *9*, 11212−11223. Ref. [66] Copyright (2017) American Chemical Society.

A physically and chemically self-repairing surface was also fabricated with a fluorinated polyurethane elastomer (FPU) and hydrophobic octa(1H,1H,2H,2H-heptadecafluorodecyl)octa-silsesquioxane by substrate surface spraying [66]. It was shown that the superhydrophobicity of the FPU/FD-POSS surface could be recovered after abrasion, scratching, burning, plasma-cleaning, sonication, chemical, and mechanical treatment. The most durable coatings contained 15 wt.% FD-POSS. Due to the migration of FD-POSS to the surface during heating (resulting in energy reduction caused by the presence of fluorine atoms) and because of the elastomeric nature of FPU (T_g_ ≪ room temperature), the coating was able to self-regenerate both chemically and physically. The time required to fully restore the superhydrophobic properties decreased with increasing temperature, as expected for a diffusion-controlled process. Thermal regeneration of the low surface energy caused by FD-POSS migration was accompanied by partial texture recovery (Figure 4). A control sample of commercially available Desmophen 670BA resin doped with octa-isobutylpoly-polyhedral oligomeric silsesquioxane (IB-POSS) was also shown to exhibit self-healing and superhydrophobic properties, despite the absence of any fluorine components [66]. Similarly, the incorporation of OH-functionalized POSS into typical acrylic-melamine clearcoats effectively increased their scratch resistance and hardness [67]. This effect was attributed to the increased density of crosslinks in the resulting nanocomposite coatings that exhibited better healing ability during heat treatment compared to the unmodified clearcoat. This was explained by physical H-bond type interactions formed in the vicinity of POSS cages. Healing increased up to a sample containing 3.8 wt.% POSS in the formulation and then decreased. Additionally, POSS additives did not have any negative effect on the optical quality of clearcoats. The only drawback of the presence of octahedral silsesquioxanes was the appearance of some irremovable cracks at higher POSS content. This effect could be attributed to the high degree of crosslinking and increased T_g_ of these clearcoats.

Crosslinking of pH-responsive polyurethane (pH-PU) and fluorinated octavinyl polyhedral silsesquioxane (F-OV-POSS) by UV curing resulted in fabric coatings with durable superhydrophobicity and self-cleaning ability under harsh conditions (mechanical damage, seawater, and UV-irradiation) [68]. Protonation and deprotonation of the pH-PU structure make the wettability of the fabric coating, its superhydrophobicity, and underwater superoleophobicity controllable by changing the pH value. The damaged surfaces could regain their particular wettability by heating, which facilitated the migration of F-OV-POSS fluorocarbon chains to the outer surface of the coatings. Such self-healing material with switchable wettability and durable self-cleaning and oil/water separating properties can be used in energy conservation and sustainability applications.

**Figure 5:**
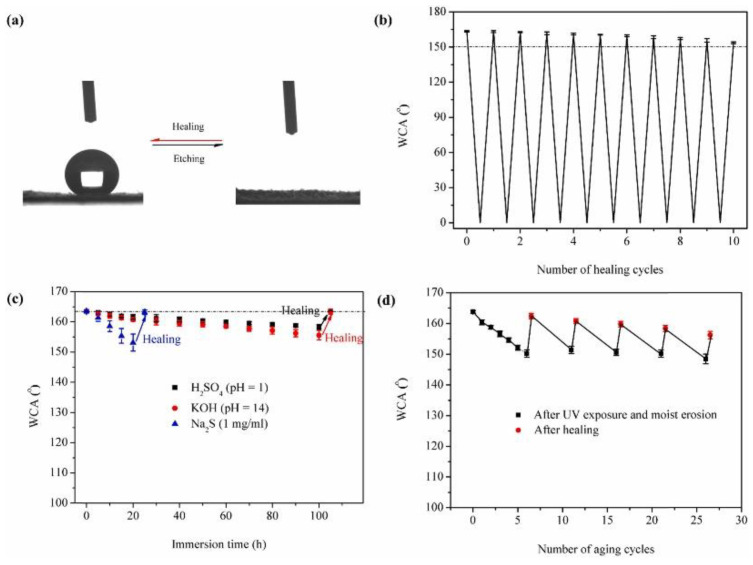
Wettability change of hybrid cotton fabric after oxygen plasma etching and the following self-repairing (**a**) with the number of O_2_ plasma etching (**b**), chemical treatment time (**c**), and UV aging and moist erosion cycles (**d**). Reprinted from *Colloids Surf. A Physicochem. Eng. Asp.* 2021, *6305*, Li, H.; Miao, S.; Chen, W.; Yang, X.; Li, M.; Xing, T.; Zhao, Y.; Chen, G., Durable superhydrophobic and oleophobic cotton fabric based on the grafting of fluorinated POSS through silane coupling and thiol-ene click reaction, 127566. Ref. [69] Copyright (2021), with permission from Elsevier.

Cotton fabrics with durable superhydrophobic and oleophobic properties were also obtained by coupling (heptadecafluoro-1,1,2,2-tetradecyl)trimethoxysilane and 3-mercaptopropyltriethoxysilane (MPTES) directly on fibers and then treating them with UV light to induce a thiol-ene click reaction between octavinyloctasilsesquioxane and thiol groups of MPTES and 1H,1H,2H,2H-perfluorododecyl-1-thiol [69]. The thus prepared coated cotton fabric exhibited not only superhydrophobicity and high oleophobicity, but also the self-healing ability and good abrasion and erosion resistance (Figure 5).

### 2.3. Silsesquioxane Structures Inserted in Main Backbones of Self-Healing Polymers

**Figure 6:**
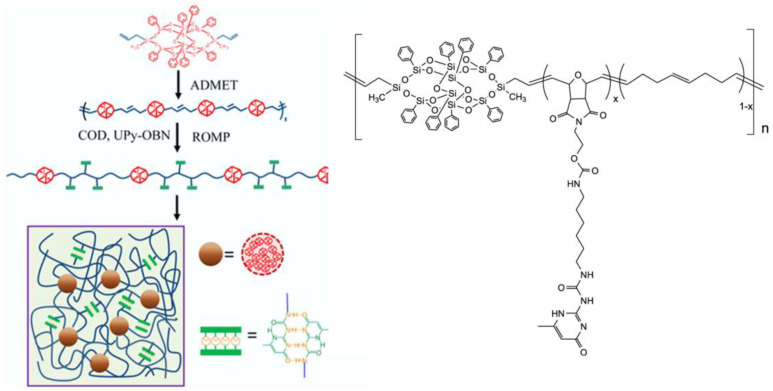
Synthesis of hybrid organic-inorganic P(DDSQ-COD-co-UPy) copolymers and illustration of DDSQ phase separation within the polymer matrix. Reprinted with permission from *Macromolecules* 2020, *53*, 7119–7131. Ref. [70] Copyright (2020) American Chemical Society.

**Figure 7:**
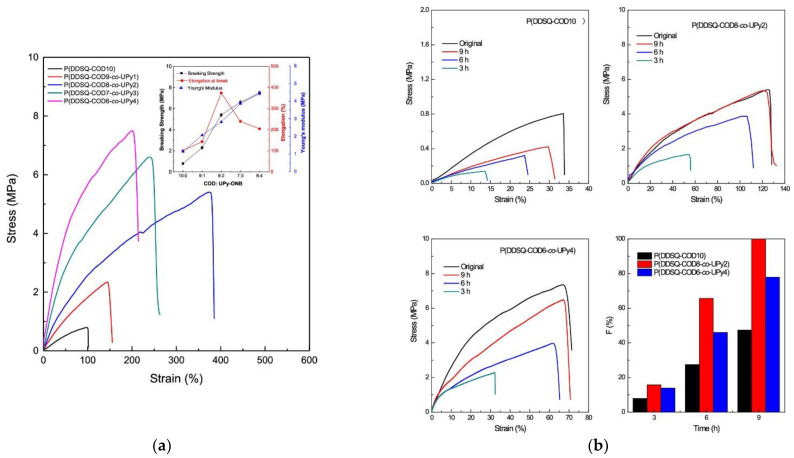
(**a**) Stress–strain curves of P(DDSQ-COD-co-UPy)s; (**b**) Stress–strain curves as functions of self-healing time. Reprinted with permission from *Macromolecules* 2020, *53*, 7119−7131. Ref. [70] Copyright (2020) American Chemical Society.

**Figure 8:**
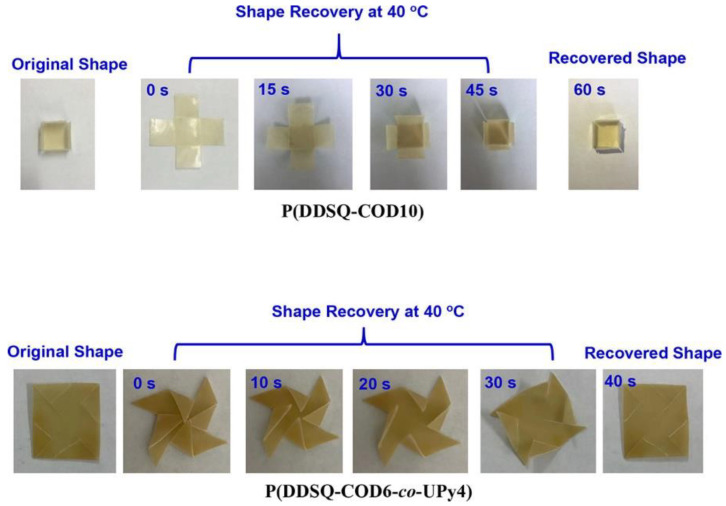
Shape recovery of hybrid polymers P(DDSQ-COD10) and P(DDSA-COD6-*co*-UPy4) at 40 °C. Reprinted with permission from *Macromolecules* 2020, *53,* 7119–7131. Ref. [70] Copyright (2020) American Chemical Society.

Double-decker silsesquioxanes (DDSQ) are hybrid molecules of a special type that consist of eight silsesquioxane segments (RSiO_3/2_) and two difunctionalized siloxane units (R,R’SiO_2/2_) located on opposite sides of the inorganic cage. It allows for their use as co-monomers in various polymerization processes. Molecules of 3,13-diallyl DDSQ were used to form a macromolecular chain transfer agent for ring-opening metathesis polymerization (ROMP) via acyclic diene metathesis (ADMET) [70]. DDSQ was copolymerized with 4-(2-hydroxyethyl)-10-oxa-4-azatricyclo-[5.2.1.02,6]dec-8-ene-3,5-dione-2-ureido-4[1H]-pyrimidinone as a co-monomer via ROMP to regulate specific intermolecular interactions in the polymer matrix. Aggregation of DDSQ into a specific nanophase (average size of inclusions ~ 20−30 nm) made the synthesized organic−inorganic copolymers heterogeneous at the nanometer scale (Figure 6). The nanodomains became net points of physically crosslinked networks, and consequently, the copolymers behaved as crosslinked elastomers with thermally induced shape memory properties. The hybrid materials exhibited good self-healing properties and ductility in the solid state (380% elongation at break at room temperature), which was due to the presence of supramolecular dimers formed by 2-ureido-4[1H]-pyrimidinone groups connected by quadruple hydrogen bonds and their dynamic exchange (Figure 7). Moreover, the self-healing properties favorably influenced shape memory in the solid polymers (Figure 8).

**Figure 9:**
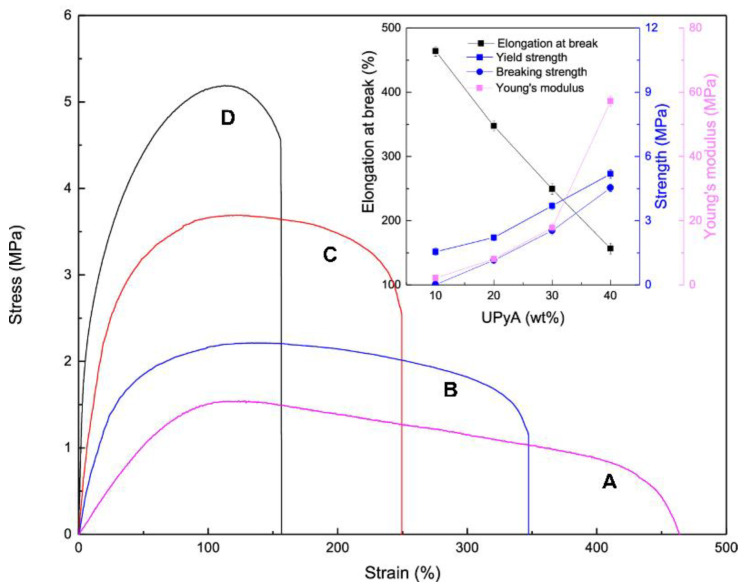
Stress-strain curves and analysis of elongation at break changes with the content of UPy for (**A**) DDSQ[P(BA-*co*-UPyA10]_2_, (**B**) DDSQ[P(BA-*co*-UPyA20]_2_, (**C**) DDSQ[P(BA-*co*-UPyA30]_2_ and (**D**) DDSQ[P(BA-*co*-UPyA40]_2_. Reprinted with permission from *ACS Appl. Polym. Mater*. 2020, *2*, 3327–3338. Ref. [72] Copyright (2020) American Chemical Society.

Analogously, 3,13-dihydroxyl-DDSQ were used as main chain extenders in hybrid linear segmented poly(urea urethanes) (PUUs) with hindered urea bonding, which were prepared by copolymerization of 4-butanediol and N,N′-di-tert-butylethylenediamine [71]. The materials were microphase-separated with POSS aggregated into microdomains (size of ~10–20 nm). The thermomechanical properties of the organic-inorganic PUUs were improved. The SHM also exhibited shape memory properties and plasticity in the solid state at room temperature due to the dynamic exchange of hindered urea bonds. The self-healing ability of PUUs was not significantly affected by the reinforcement with POSS nanodomains. The synthesis of P(DDSQ-COD-*co*-UPy) copolymers with DDSQ chain extenders enabled the introduction of both inorganic silsesquioxane structures and UPy units into the main chains of nonpolar polyolefins to improve their functional properties [70]. The self-healing of P(DDSQ-COD8-*co*-UPy2) was demonstrated by placing two fracture surfaces in close contact and annealing them at room temperature for 24 h. As expected, the self-repairing depended on the content of UPy moieties and on the increment of healing time. Tensile tests showed that the mechanical properties were restored up to 100%, and the healed specimens had good tensile strength and elongation at break (Figure 9). As the healing time increased, the stress-strain curves became increasingly similar to those of the uncut specimens, indicating a gradual recovery of the tensile mechanical properties. However, the extent of the recovery was not monotonously consistent with the increase in UPy content, which was due to the restriction of the movement of polymer backbone segments by the quadruple hydrogen bond clusters. This was evidenced by a corresponding increase in the glass transition temperature. Consequently, the dynamic exchange of quadruple hydrogen bonds was slowed down. The compromise of these two opposing tendencies was an optimal restoration of mechanical properties.

Similar results were obtained for self-healing DDSQ[P(BA-*co*-UPyA)] copolymers prepared by RAFT copolymerization of butyl acrylate (BA) with 2-ureido-4[1H]-pyrimidinone acrylate (UPyA) using trithiocarbonate derivative of 3,13-dihydroxypropyl DDSQ [72] or polyureas obtained with the use of α,ω-diaminoterminated poly(propylene oxide), carbon dioxide and 3,13-dianilino DDSQ [73]. The hybrid polyureas were microphase-separated with DDSQ cages aggregated into spherical microdomains with diameters of 50–120 nm, which were assigned as the cause of the crosslinked elastomeric behavior of the material. The silsesquioxane content had a significant effect on the self-healing properties based on the dynamic exchange of multiple hydrogen bonds. In the case of DDSQ[P(BA-*co*-UPyA)] the double-decker silsesquioxanes aggregated into much smaller microdomains with dimensions of 10–20 nm. All the studied samples showed high elongation at break, which increased with the content of UPyA groups in the material, e.g., 450% for DDSQ[P(BA-*co*-UPyA10)]_2_ and 350% for DDSQ[P(BA-*co*-UPyA20)]_2_. The UPyA content also affected the shape recovery rate—the higher it was, the faster the samples reformed (shape recovery time was 85 s for DDSQ[P(BA-*co*-UPyA10)]_2_, 74 s for DDSQ[P(BA-*co*-UPyA20)]_2_, 65 s for DDSQ[P(BA-*co*-UPyA30)]_2_ and 48 s for DDSQ[P(BA-*co*-UPyA40)]_2_).

**Figure 10:**
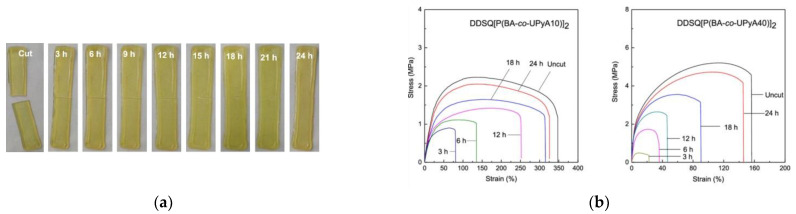
(**a**) DDSQ[P(BA-*co*-UPyA40)]_2_ samples during self-healing tests at 40 °C; (**b**) Stress−stain curves for the evaluation of the self-healing properties of DDSQ[P(BA-*co*-UPyA10)]_2_ and DDSQ[P(BA-*co*-UPyA40)]_2_ at 40 °C. Reprinted with permission from *ACS Appl. Polym. Mater.* 2020, *2*, 3327–3338. Ref. [72] Copyright (2020) American Chemical Society.

The self-healing properties were tested by cutting the samples into two halves and then contacting them at elevated temperatures (40 °C). The incision in the material gradually decreased and disappeared completely after about 15 h (Figure 10a). Tests of mechanical properties of the healed samples confirmed almost complete regeneration after about 24 h (Figure 10b). The rate of self-regeneration depended on the segmental mobility and decreased as the number of quadruple hydrogen bonds increased. At the same time, an inverse relationship was observed for the degree of self-healing, with the highest effectiveness of healing exhibited by DDSQ[P(BA-*co*-UPyA40)]_2_.

## 3. POSS Grafted to Polymers through Dynamic Bonds

**Figure 11:**
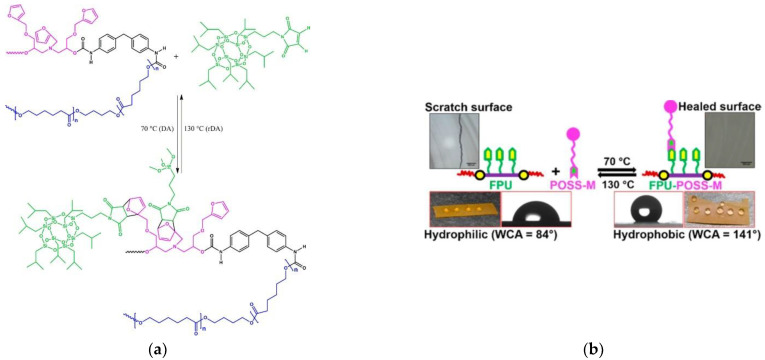
(**a**) Dynamic DA process in FPU-POSS-M and (**b**) changes in physical properties as a result of self-healing. Reprinted with permission from *Macromolecules* 2018, *51*, 4770–4781. Ref. [74] Copyright (2018) American Chemical Society.

**Figure 12:**
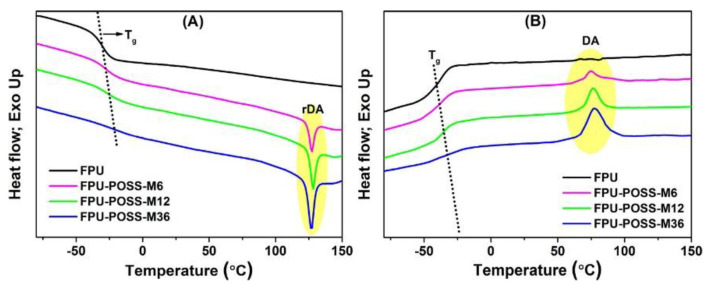
DSC heating (**A**) and cooling (**B**) thermograms of native FPU and hybrid composites FPU-POSS-M6, FPU-POSS-M12, and FPU-POSS-M36. Reprinted with permission from *Macromolecules* 2018, *51*, 4770–4781 [74] Copyright (2018) American Chemical Society.

**Figure 13:**
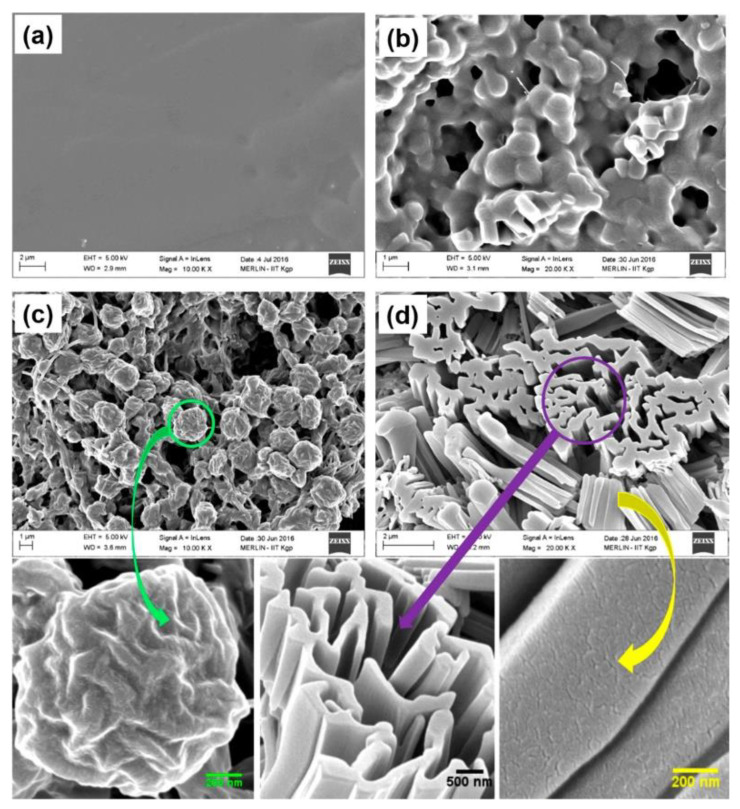
FESEM images of (**a**) native FPU and hybrid composites: (**b**) FPU-POSS-M6, (**c**) FPU-POSS-M12, and (**d**) FPU-POSS-M36. Reprinted with permission from *Macromolecules* 2018, *51*, 4770–4781 [74] Copyright (2018) American Chemical Society.

Although the presence of POSS or DDSQ can cause significant changes in the polymer systems described in the previous section, these hybrid molecules can also take a more active role, affecting the self-healing properties of the materials by forming dynamic bonds. The actual effect of functionalized silsesquioxanes on the self-repairing process in hybrid materials can be achieved by reversibly linking these nanoscale molecules to the polymer backbone. POSS molecules containing maleimide and isobutyl side groups (POSS-MI) were used in the synthesis of a new class of hydrophobic self-healing polyurethane-POSS hybrids based on the dynamic furan-maleimide Diels-Alder “click” reaction (Figure 11) [74]. The obtained ultrahydrophobic hybrid material (FPU-POSS-MI) with improved thermal stability, mechanical and nanomechanical properties, and good adhesion strength, can be used for specialty coatings. The structure of FPU-POSS-MI was confirmed by FT-IR, ^1^H NMR, and GPC analyses, while its thermoreversible nature was demonstrated by DSC studies. DSC thermograms of FPU and FPU-POSS-MI showed (Figure 12) that the glass transition temperature values obtained from the heating and cooling thermograms agreed well with each other. A gradual increase in T_g_ and the enthalpy of the DA (exothermic peak; *T*_DA_ ~75 °C) and retro-DA (endothermic peak; *T*_rDA_ ~127 °C) reactions were observed with increasing POSS-MI content, which is due to the increasing effect of rigidity of the polymer chains. The hybrid materials showed a significant increase in the wetting angle from 84° to 141.3°, owing to the combined effect of grafting of hydrophobic POSS-MI units and increased micro- and nanoscale surface roughness. There was no change in the WCA values after the samples were heated to rDA temperature (130 °C) and then annealed for another 15 h at 70 °C. The surface structure was evaluated by atomic force microscopy (AFM) and field emission scanning electron microscopy (FESEM). An interesting sphere-like morphology with small nanopatterns on the surface was observed on the FESEM micrographs (Figure 13). With increasing POSS-MI content, the sphere-like morphology changed to cauliflower-like structures with multiple petals of 20−100 nm width, and then to a combination of lamellar and folded-lamellar morphologies with large air pockets and increased roughness.

The self-healing characteristics of this organic–inorganic hybrid SHM were evaluated by a tensile test and also by monitoring the crack disappearance by optical microscopy after annealing at 130 °C for 3 h, then cooling to 70 °C and maintaining at this temperature for 15 h. Heating at 130 °C caused cleavage of the DA adducts. The bonds reconnected via the DA reaction, which occurred at 70 °C, and the scratch was healed. In the case of FPU, the scratch remained visible after the healing process, whereas the addition of POSS-MI resulted in some enhancement of the self-healing properties even after the thermal treatment was stopped. An additional slight change in the scar could be noted. For the FPU-POSS-MI hybrids, almost no change in the surface roughness value (±3 nm) was observed after the healing process.

**Figure 14:**
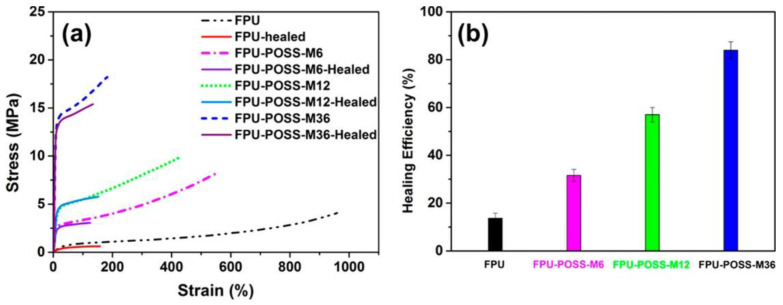
Stress−strain curves (**a**) and healing efficiency (**b**) of the native and self-repaired samples of FPU, FPU-POSS-M6, FPU-POSS-M12, and FPU-POSS-M36. Reprinted with permission from *Macromolecules* 2018, *51*, 4770–4781 [74] Copyright (2018) American Chemical Society.

The presence of POSS also improved the thermal and mechanical characteristics compared to those of pure FPU (Figure 14). The FPU-POSS-MI hybrid exhibited higher tensile strength and higher tensile modulus. Although the addition of POSS-MI increased the self-healing efficiency because of the formation of thermoreversible DA adducts, the healed samples exhibited lower elongation at break than the original specimens. Furthermore, the surface hardness of FPU-POSS-MI coated glass slides were higher than that of pure FPU and increased with the increment of POSS-MI content. Compared to FPU, the FPU-POSS-MI hybrids also exhibited better adhesion strength with metal substrates, as shown by the results of lap-shear tests. This effect was attributed to the incorporation of maleimide groups by grafting POSS-MI molecules onto furan moieties at polyurethane chains. This resulted in an increase in molecular weight, as well as an improved interaction between the metal surface and the polar maleimide functional groups. This effect was confirmed by a decrease in adhesion strength for mixtures of FPU and octaisobutyl POSS without maleimide groups.

**Figure 15:**
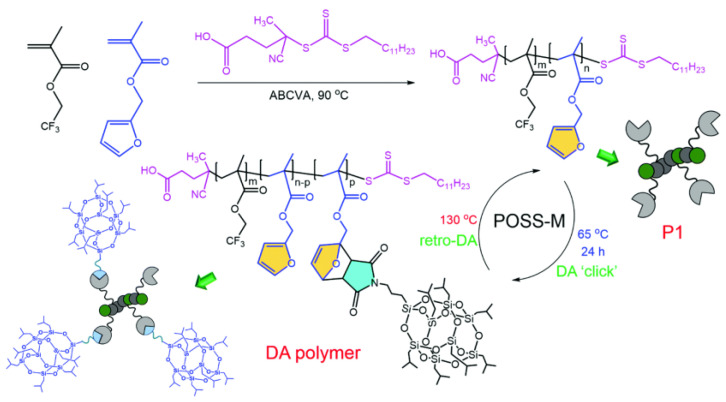
Copolymerization of FMA and TFEMA and subsequent modification of the prepared polymer with POSS-M. Reproduced from *Polym. Chem*. 2021, *12*, 876–884. Ref. [75] with permission from the Royal Society of Chemistry.

The reversible addition-fragmentation chain transfer copolymerization of furfuryl methacrylate (FMA) and 2,2,2-trifluoroethyl methacrylate (TFEMA), with silsesquioxane side units grafted by dynamic DA cycloaddition reactions (Figure 15), yielded a new class of hydrophobic functional fluoropolymers with effective self-healing properties that can be used as specialty paints and coatings [75]. The modification with POSS-MI enhances the rigidity of macromolecular chains in the methacrylate polymers, resulting in higher glass transition temperatures for DA copolymers prepared from POSS-MI (T_g_ > 90 °C). Moreover, the incorporation of hydrophobic silsesquioxane moieties significantly improved the thermal stability of the copolymers as well as the surface hydrophobicity (WCA ≈ 101° for the parent fluoro-polymer and WCA 135° for the modified polymers) due to the increased micro/nano-surface roughness.

**Figure 16:**
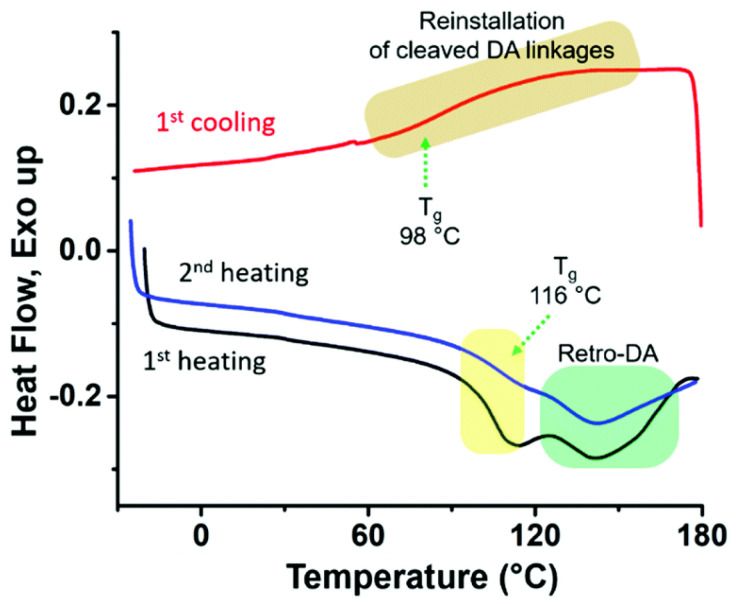
DSC traces illustrating retro-DA and DA reactions in a FMA-TFEMA-POSS hybrid. Reproduced from *Polym. Chem*. 2021, *12*, 876–884. Ref. [75] with permission from the Royal Society of Chemistry.

The thermoreversible behavior of the furan-POSS-M linkages facilitated the self-repairing of the hybrid polymethacrylates, as confirmed by DSC measurements (Figure 16) and optical microscopy analyses. The broad endotherm at the temperature region ≈ 146 °C (*T*_rDA_) in the first heating curve indicates a retro-DA reaction, whereas the exothermic peak within the temperature range of 60 °C–155 °C was attributed to the reinstallation of the cleaved DA linkages. The reappearance of the glass transition and the presence of the rDA endotherm in this temperature range in the second heating curve confirmed the reversible dynamic behavior of the DA structures in the hybrid material. The enthalpic effects observed for sample P1C with the number of reactive furan units in the copolymer chain: POSS-MI = 38:40 are higher than in the other polymers studied [ΔH_rDA_ = 10.5 J g^−1^ (1st heating curve), 5.3 J g^−1^ (2nd heating curve), ΔH_re-DA_ (9.5 J g^−1^)]. These observations were confirmed by FTIR studies.

The cut and heal tests showed that the polymers were not suitable for healing at room temperature due to the poor segmental movement of the polymer chains inhibited by POSS and the lack of reversible exchange characteristics. However, self-healing of the studied system was possible at higher temperatures. Thermal treatment of the notched sample (annealing for 5 h at 130 °C followed by annealing at 65 °C for 24 h) resulted in a fourfold reduction of the scratch width. At temperatures suitable for retro-DA reactions, segmental motion and local diffusion of polymer chains are facilitated by cleavage of DA linkages, which releases bulky molecules of POSS-MI. The structural integrity of the sample was achieved by reconnecting the cleaved functional groups at 65 °C. The self-repairing performance of DA-modified polymers was significantly improved after increasing the content of reversible DA conjugates with POSS-MI. For the P1C polymer, the relatively low repair efficiency (EH ≈ 17%) of the original parent polymer was increased to EH ≈ 78%.

**Figure 17:**
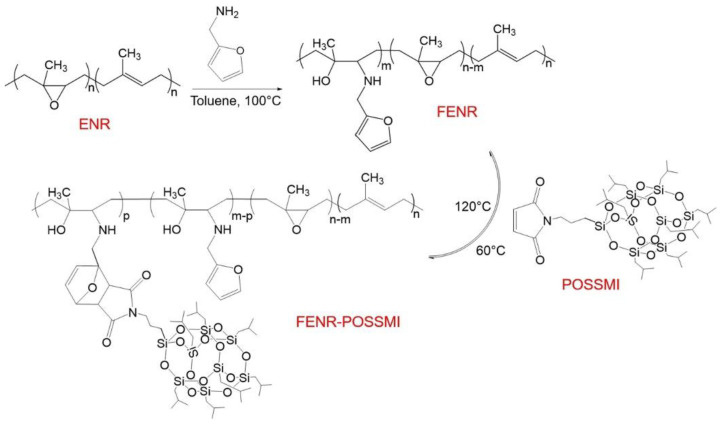
Synthesis of furfuryl modified ENR (FENR) and its thermoreversible DA ‘click’ post-modification with POSSMI. Reprinted from *Eur. Polym. J.* 2021, *146*, Raut, S.K.; Mondal, P.; Parameswaran, B.; Sarkar, S.; Dey, P.; Gilbert, R.; Bhadra, S.; Naskar, K.; Nair, S.; Singha, N.K., Self-healable ultrahydrophobic modified bio-based elastomer using Diels-Alder ‘click chemistry’, 110204, [76]. Copyright (2021), with permission from Elsevier.

The Diels-Alder reaction was also used to introduce self-healing and ultrahydrophobic features into a commercially available epoxidized natural rubber (ENR) (Figure 17) [76]. Furfuryl groups were grafted onto the backbone of the pristine elastomer and then modified by DA “click” with POSS-MI molecules. The DA furan-maleimide bonds were found to be cleaved by heating at 120 °C and re-bonded during annealing at 60 °C, as shown by DSC and FTIR analyses. The increase of T_g_ in POSS-modified ENR compared to the original sample indicates that also in this case the incorporation of bulky POSS-MI inhibits the mobility of macromolecular chains in hybrid DA elastomers. The self-healing characteristics were investigated by cut-and-heal test using optical microscopy. Due to the presence of nano-sized POSS, the DA-modified elastomer exhibits significantly better hydrophobicity (WCA > 140°), thermal stability, surface hardness, mechanical properties, and adhesion to metal substrates.

**Figure 18:**
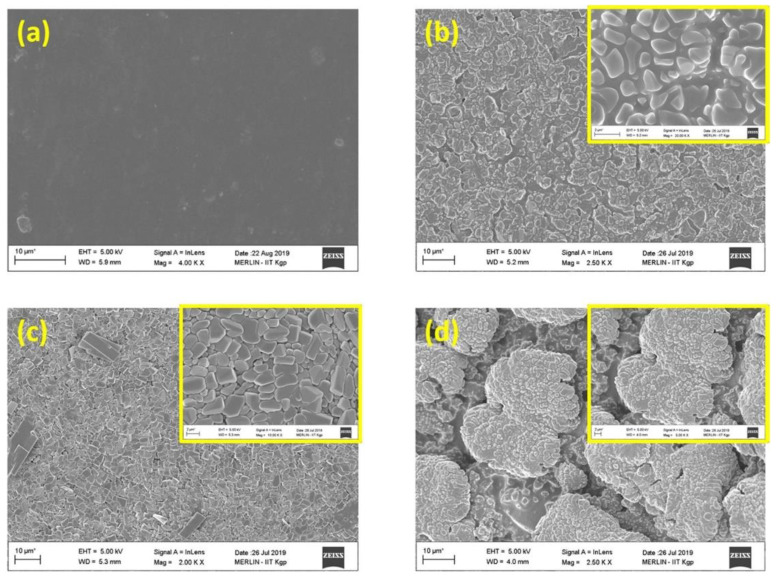
FE-SEM images of (**a**) native FENR, and FENR-POSSMI hybrids containing (**b**) FENR-POSSMI A, (**c**) FENR-POSSMI B and (**d**) FENR-POSSMI C (he ratio of furan groups to POSSMI, respectively, 27:1, 9:1, 3:1). Reprinted from *Eur. Polym. J*. 2021, *146*, Raut, S.K.; Mondal, P.; Parameswaran, B.; Sarkar, S.; Dey, P.; Gilbert, R.; Bhadra, S.; Naskar, K.; Nair, S.; Singha, N.K., Self-healable ultrahydrophobic modified bio-based elastomer using Diels-Alder ‘click chemistry’, 110204, [76]. Copyright (2021), with permission from Elsevier.

A gradual increase in surface roughness of the hybrid elastomers with increasing POSS-MI content was demonstrated on FESEM micrographs and 3D AFM images illustrating the surface morphology (Figure 18). The 0.5–5 µm “pebble-like” objects that appeared after POSS grafting resulted in an increase in surface roughness. Further increasing the load of grafted POSS-MI resulted in the formation of a three-dimensional morphology resembling lotus leaves (size ~ 20–30 µm). Importantly, the surface morphology of all the studied hybrid elastomers (and thus their hydrophobicity) was almost preserved after the self-healing process. The inclusion of bulky POSS-MI molecules affects the overall mobility of the DA elastomer, which hinders healing at room temperature. As demonstrated in previous examples of hybrid materials operating on DA chemistry, the dynamic behavior of furan-maleimide DA conjugates at *T*_rDA_ plays a significant role in enabling segmental movement of polymer chains through the release of POSS-MI molecules. This effect was confirmed by the lap-shear test. The increased segmental movement of polymer chains caused by the release of POSS-MI facilitated the flow of the released polymer chains (T_g_ < *T*_rDA_ because furfuryl groups acted as a plasticizer). Consequently, the healing process was enhanced by increased mobility of macromolecular chains. Again, the healing performance of DA elastomers improved significantly with increasing POSS-MI content.

## 4. Self-Healing Crosslinked Materials Based on Dynamic Interactions with Octafunctional POSS

**Figure 19:**
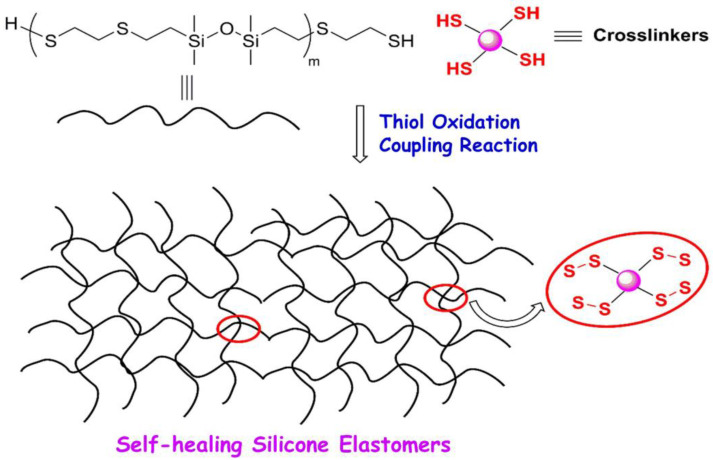
Synthesis of disulfide-linked SEs. Reprinted under the terms and conditions of the Creative Commons Attribution (CC BY) license from *Polymers* 2021, *13*, 3729 [79].

The application of SHM, which can potentially be used as coatings, low-electrical insulating materials for microelectronics, or automotive clearcoats, is related to the type of polymer matrix. For example, self-healing silicones, in addition to their high thermal stability, hydrophobicity, biocompatibility, and electrical resistance have recently received special attention, especially due to new emerging applications in stretchable electronics [77,78]. Molecules of functionalized octa(3-mercaptopropyl)silsesquioxane (POSS-SH) have been used as crosslinking agents for self-healing disulfide-linked silicone elastomers (SEs) based on thiol-terminated sulfur-containing heterochain polysiloxanes (P-SHs) (Figure 19) [79]. Their efficiency in the thiol oxidation coupling reactions was compared to that of pentaerythritol tetrakis(β-mercaptopropionate) (PETMP) and poly[(mercaptopropyl)methylsiloxane] (PMMS). The silicone elastomers were self-healed via reversible disulfide bonds with EH >70% by heating at 150 °C for 2 h or UV irradiation for 30 min. The structure of the crosslinked silicones was determined by FTIR spectroscopy as the peak at ~2540 cm^−1^ attributed to ν(SH) disappeared and a new peak assigned to ν(S–S) vibrations appeared at 472 cm^−1^, resulting from the oxidation of -SH groups.

The mechanical properties of crosslinked SEs can be adjusted by changing the amount and type of components used as crosslinking agents. Too low amount of crosslinkers resulted in poor elastomeric properties. Using POSS and higher amounts of PETMP as linkers between macromolecules led to the formation of hard SEs with numerous cracks. POSS-SH is a bulky molecule and, therefore, free rotation of bonds in the formed crosslinked network is difficult. Unlike molecular crosslinkers, such as PETMP and POSS-SH, PMMS with high molecular weight cannot effectively bond the polysiloxane matrix. The reason is the crosslinking between PMMS chains, leading to unsuccessful hybrid network formation. Moreover, POSS-SH could provide more crosslinking sites than PETMP. As a result, more rigid and dense networks were formed, which was reflected in the mechanical properties. As the amount of POSS-SH increased, the elongation at break gradually decreased from 545% to 85%, while the change of the tensile strength trend was rather irregular, and the sample with a moderate amount of crosslinking agent showed the highest tensile strength (0.23 MPa).

**Figure 20:**
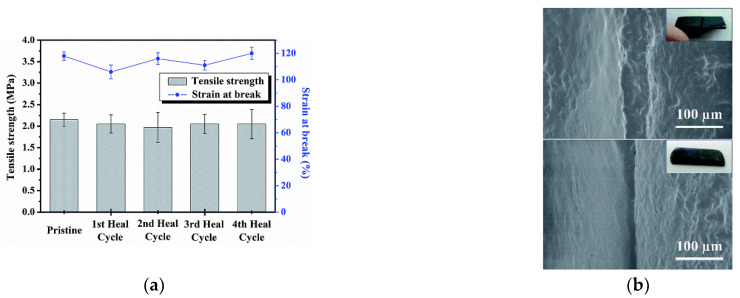
(**a**) Stress-strain curves for pristine and healed POSS-U/PCL-A samples; (**b**) Comparison of SEM micrographs of POSS-U/PCL-A films after 2 h (top photo) and 24 h (bottom) healing periods at room temperature (inserts—photographs of the mended samples). Reproduced from *RSC Adv*. 2015, *5*, 90466–90472 [80] with permission from the Royal Society of Chemistry.

Another method for obtaining hybrid polymer coatings with self-healing properties is based on the use of complementary interactions between nucleobase pairs. A novel system containing adenine end-capped three-arm polycaprolactone oligomer (PCL-A) and POSS bearing eight uracil groups (POSS-U) was successfully used to prepare stable, physically crosslinked structures via the formation of complementary uracil–adenine (U–A) pairings [80]. POSS-U formed thermo-reversible hydrogen bonded complexes with PCL-A, that were sensitive to temperature changes. Various POSS-U/PCL-A blend ratios (10/1; 3/1; 1/1) were used to prepare thermo-responsive films with different mechanical properties. It was found that a high POSS-U content promoted plastic behavior (Young’s modulus of 30.3 MPa, elongation at break of 43.1% for POSS-U/PCL-A at 10/1) whereas larger amounts of PCL-A resulted in the domination of elastic behavior (Young’s modulus = 9.6 MPa, elongation at break of 587.6% for POSS-U/PCL-A at 1/1). The prepared films exhibited solid-gel transition from an elastomeric solid to a viscous liquid state upon heating to 120 °C and were capable of remodeling under mild temperature conditions. During repeated stress–strain tests, the samples displayed comparable mechanical properties with a tensile strength of 2.05 MPa and a strain at break of 114% (Figure 20a). Examination of the self-healing behavior of POSS-U/PCL-A films showed that approximately 32% of the original rupture strain was recovered after 12 h at room temperature, and the maximum performance (92%) was reached after 24 h (Figure 20b). The mild self-healing conditions and relatively short time required for the self-repair make POSS-U/PCL-A films and the use of complementary base pairing very attractive for potential applications (e.g., as wound healing materials).

**Figure 21:**
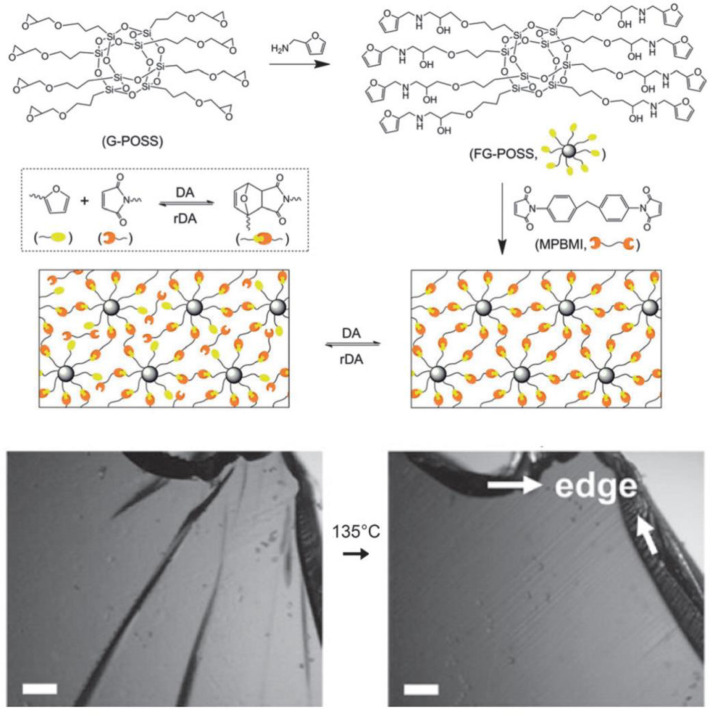
Cracked and self-healed sample after thermal treatment (30 min at 135 °C) with a DA and retro-DA reaction mechanism scheme (scale bar 1 mm). Reproduced from *Chem. Commun*. 2013, *49*, 6755–6757 with permission from the Royal Society of Chemistry [81].

Self-repairing hybrid coatings can be also formed from mixtures of functionalized POSS and complementarily reactive small molecules. For example, a tough, transparent, and self-healing POSS nanocomposite was prepared by a thermally reversible Diels–Alder reaction between furan-end octafunctionalized POSS (FG-POSS) and bismaleimide molecules (Figure 21) [81]. The DA reaction progress was monitored by ^1^H NMR, UV, and FT-IR spectroscopy. The degree of conversion of the epoxy groups was found to be about 98%. The formation of DA bonds between furan and maleimide groups was indicated by shifting the carbonyl doublet characteristic IR peak (1770 cm^−1^) and the imide C=O stretching vibrations (1709 cm^−1^) to higher wavenumbers of 1774 and 1713 cm^−1^, respectively. The conversion of maleimide to DA adduct could be also observed as a decrease in absorption at about 300 nm due to the loss of O=C–C=C–C=O conjugation.

Good transparency of the crosslinked material indicated the molecular level of FG-POSS dispersion in the rigid nanocomposite at room temperature. The compressive modulus value was 1.0 GPa and the yield strength (118 MPa) was comparable to those obtained for commercially available crosslinked epoxy resins (102–170 MPa) [82]. From the results of the DSC analysis, it was noted that the retro-DA reaction occurs in the temperature range 94 °C−210 °C. As indicated by the presence of a broad endothermic peak only in the first heating trace, the retro-DA reaction in the nanocomposite occurred only when the healing conditions were suitable to allow the reattachment of furan and maleimide moieties. Furthermore, the self-healing temperature should not exceed 180 °C because at elevated temperatures polymerization of free maleimide molecules can occur as an undesired side reaction leading to the formation of irreversible bonds [83].

The cracked sections of the POSS nanocomposite could be thermally repaired. After heating at 135 °C for 30 min and then cooling, the cracks were successfully healed without leaving a scar (Figure 21). Heating at 150 °C for 30 min also healed the cracks but not the surface scar, which remained visible due to the loss of material. However, self-healing occurred only when the damaged parts were in very close contact with each other. Furthermore, the retro Diels-Alder reaction was found to become more difficult in successive heating-cooling cycles, which was also confirmed by DMA measurements. The storage modulus and the rate of its decrease were found to diminish with each successive heating-cooling cycle (measurements at 38 °C; 1st heating—1870 MPa, 2nd—1310 MPa, 5th—1120 MPa). The increased difficulty in the retro-DA process was also confirmed by the decrease in material rigidity and the associated change in the tan δ peak value from 85 °C for the original sample to 116 °C in the 5th heating scan.

**Figure 22:**
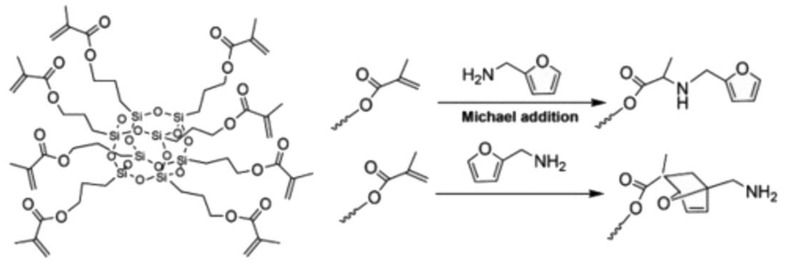
Chemical structures and reactions between MMA-POSS and FA. Reprinted from *Compos. Sci. Technol*. 2015, *118*, Chuo, T.-W.; Liu, Y.-L., Preparation of self-healing organic-inorganic nanocomposites with the reactions between methacrylated polyhedral oligomeric silsesquioxanes and furfurylamine, 236–243, [84]. Copyright (2015), with permission from Elsevier.

**Figure 23:**
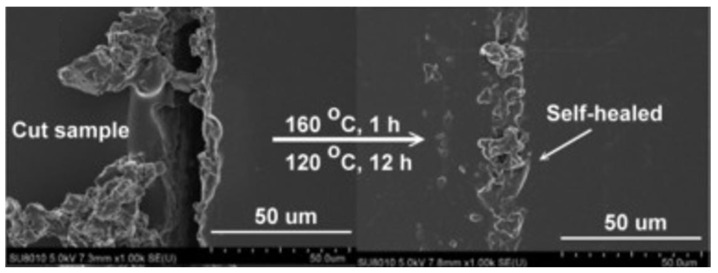
SEM micrographs of MMA-POSS/FA films before and after self-healing at 160 °C for 1 h and at 120 °C for 12 h. Reprinted from *Compos. Sci. Technol*. 2015, *118*, Chuo, T.-W.; Liu, Y.-L., Preparation of self-healing organic-inorganic nanocomposites with the reactions between methacrylated polyhedral oligomeric silsesquioxanes and furfurylamine, 236–243, [84]. Copyright (2015), with permission from Elsevier.

A crosslinked, thermally stable (up to 300–350 °C) nanohybrid material (MMA-POSS/FA) was prepared using commercially available octafunctional POSS containing methacrylate groups (MMA-POSS) and furfurylamine (FA) [84]. In the system studied, both the Michael addition reaction of MA-amine and the Diels-Alder reaction of MA-furan took place between the two monomers (Figure 22). The interactions between the POSS units resulted in the formation of well-defined self-organized lamellar structures with a spacing width of about 455 nm. Residual methacrylate groups make these materials amenable to crosslinking. Furthermore, the nanohybrids exhibited thermal self-healing ability based on the reversible formation of MA-furan DA adducts that may have potential applications in self-healing coatings and low-electrical insulating materials for microelectronics. Self-regeneration tests were performed with a viscous MMA-POSS/FA mixture, which was coated onto silicon wafers and cured at 210 °C for 1 h. To determine the most appropriate self-healing conditions, the cut marks were examined by SEM. Complete disappearance of the cuts was observed after heat treatment at 160 °C for 1 h and then at 120 °C for 12 h (Figure 23). Unfortunately, mechanical testing could not be performed due to the high brittleness of the studied materials.

**Figure 24:**
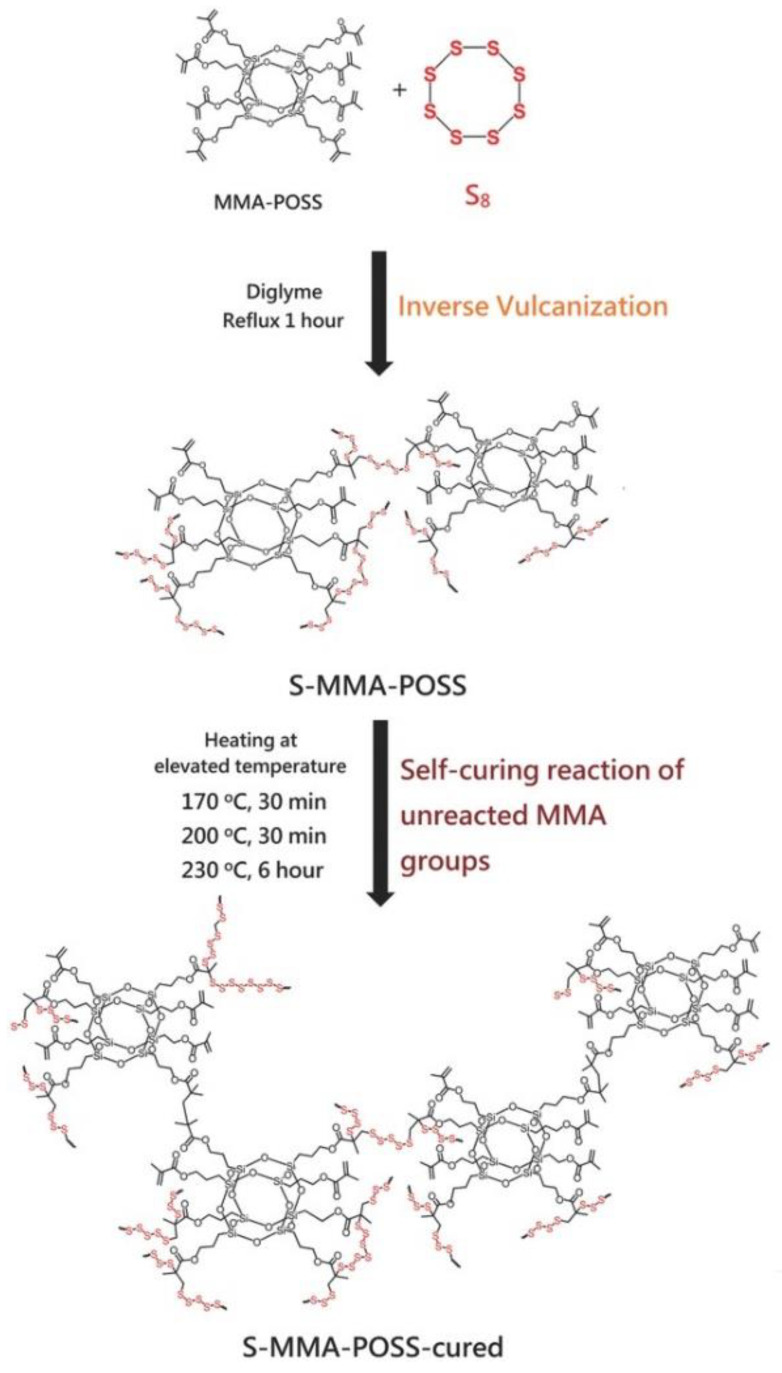
Synthetic routes for preparation of reactive hybrid S-MMA-POSS. Copyright (2017) Wiley. Used with permission from (Lin, H.-K.; Liu, Y.-L., Reactive Hybrid of Polyhedral Oligomeric Silsesquioxane (POSS) and Sulfur as a Building Block for Self-Healing Materials, *Macromol. Rapid Commun*. 2017, *38*, 1700051; WILEY-VCH Verlag GmbH & Co. KGaA, Weinheim) [85].

**Figure 25:**
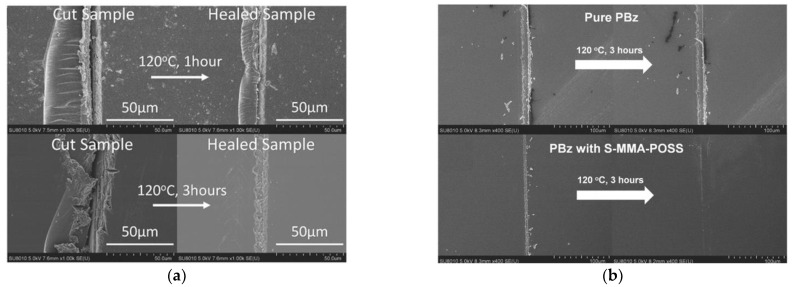
Comparison of (**a**) SEM micrographs of POSS-MMA-S samples before and after the self-healing process at 120 °C for 1 h and 3 h; (**b**) self-healing abilities of pristine PBz and PBz/POSS-MMA-S films. Copyright (2017) Wiley. Used with permission from (Lin, H.-K.; Liu, Y.-L., Reactive Hybrid of Polyhedral Oligomeric Silsesquioxane (POSS) and Sulfur as a Building Block for Self-Healing Materials, *Macromol. Rapid Commun*. 2017, *38*, 1700051; WILEY-VCH Verlag GmbH & Co. KGaA, Weinheim) [85].

**Figure 26:**
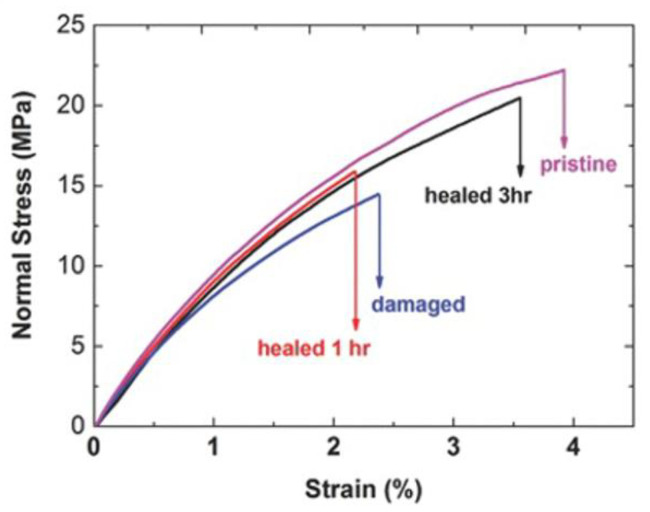
Self-healing effectiveness of thermally cured S-MMA-POSS samples evaluated with stress–strain tests. Copyright (2017) Wiley. Used with permission from (Lin, H.-K.; Liu, Y.-L., Reactive Hybrid of Polyhedral Oligomeric Silsesquioxane (POSS) and Sulfur as a Building Block for Self-Healing Materials, *Macromol. Rapid Commun*. 2017, *38*, 1700051; WILEY-VCH Verlag GmbH & Co. KGaA, Weinheim) [85].

A new type of self-healing material was obtained by reacting MMA-POSS methacrylate and elemental sulfur (S_8_) by reverse vulcanization (Figure 24) [85]. A reactive and functional hybrid macromolecule (S-MMA-POSS) was prepared, which can serve as an effective building block for imparting self-healing ability to thermally crosslinked nanocomposites in self-curing and co-curing reactions with conventional thermosetting resins. S-MMA-POSS exhibited high mid-infrared transparency due to the presence of polysulfide linkages. The relatively low bond dissociation energy and low temperatures required for disulfide bond exchange may be an interesting alternative to self-healing materials based on the DA reaction [86]. The self-repairing properties were investigated for cut S-MMA-POSS films, which were heat treated at 120 °C for 1 h. Under these conditions, the disulfide bonds were partially broken, making the molecular chains more amenable and allowing the cuts to heal. The S-S disulfide bonds restored the crosslinked structure of the sample upon cooling to room temperature. However, it was found that 1 h of heat treatment allowed only partial self-healing of the sample (Figure 25a). The mechanical properties of the original S-MMA-POSS films showed a tensile strength of 22.0 MPa and 3.7% elongation at break, while for the self-healing samples it was reduced to, respectively, 13.4 MPa and 1.7%. Increasing the heat treatment time to 3 h allowed the regeneration efficiency based on tensile strength to be increased to about 91% (tensile strength—20.1 MPa and elongation at break—3.5%) (Figure 26). The obtained S-MMA-POSS were also applied as additives (25 wt.%) to a benzoxazine-containing polymer (PBz) in order to test the possibility of their use as functional additives imparting self-healing ability to crosslinked polymers. It was confirmed that under identical self-healing conditions, the PBz/S-MMA-POSS cut sample exhibited significant self-healing properties, while PBz showed no self-healing ability (Figure 25b). Moreover, POSS can act not only as a self-healing agent for resins, but also potentially improve their thermal and mechanical properties.

An alternative method to obtain self-healing hybrid systems is based on the use of metal−ligand (M−L) interactions. The thermodynamic and kinetic properties of M−L complexes may enable the synthesis of new hybrid materials with tunable mechanical properties [87]. Moreover, M−L interactions are less sensitive to moisture than hydrogen bonds, which also increase the possibility of their practical application. Due to the high association constants and the fact that the M−L complexes usually form ionic clusters, external energy, such as heat [88] or light [89] is necessary for the reversible dissociation of the M−L complexes to induce self-healing. The most popular self-healing M−L systems involve nitrogen-based ligands (e.g., terpyridines, imidazole, histidine).

**Figure 27:**
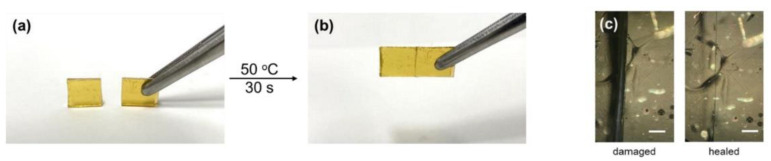
(**a**) POSS-B/ZnCl_2_ sample cut into two pieces; (**b**) rejoined pieces after 30 s at 50 °C; (**c**) images of sample before (left) and after (right) self-healing process. Scale bar—500 μm. Reproduced from *Mater. Chem. Front.* 2020, *4*, 2655–2664 [90] with permission from the Royal Society of Chemistry.

An interesting example of the use of M-L interactions in the synthesis of new self-healing hybrid materials was presented for a zinc/imidazole-based system that could be used as a hard coating [90]. ZnCl_2_ was used as a metal salt due to its good thermal stability and fairly weak interactions between zinc and chloride ions [91] which reduced the temperature required for the self-healing process. Dual-functionalized POSS bearing imidazole and n-alkyl groups (2-methylthioethyl—POSS-M, butyl—POSS-B, and undecyl groups—POSS-U) in a single arm were synthesized by thiol–epoxy click reaction followed by esterification. Synthesis of materials with different n-alkyl chain lengths yielded materials with tunable flexibility which was confirmed by the results of DSC studies. The observed glass transition temperature values decreased significantly for longer alkyl chains (T_g_ = −29 °C, −35 °C, and −51 °C, for POSS-M, POSS-B, and POSS-U, respectively). All materials were thermally stable up to 250 °C. Further heating caused rapid decomposition of the organic functional groups. Based on the TGA results, the molar ratio ZnCl_2_/imidazole was estimated to be 0.71, 0.51, and 0.55 for POSS-M, POSS-B, and POSS-U, respectively. The higher ZnCl_2_ content in the former case was explained by its complexation not only with imidazole moieties but also the formation of chelates with sulfur atoms. The latter effect also contributed to the best mechanical properties of POSS-M (Young’s modulus = 2.13 GPa). As expected, the type of alkyl side chains also played a significant role in the self-healing properties of the nanocomposites. This ability was studied for samples cut into two pieces and then thermally healed at 50 °C. It was found that the POSS-B sample was successfully repaired after only 5 s, leaving only a small scar (Figure 27). POSS-B and POSS-U exhibited an 80% mechanical self-healing ratio (in comparison to the properties of the original samples) after healing for 24 h. At the same time, POSS-M was only self-healed in 46% due to the presence of thioether groups, which slowed down the ligand exchange between zinc and imidazole moieties. The time required for self-healing of the samples was significantly shorter when increasing the healing temperature (98% regeneration after 10 min at 80 °C).

In addition to the effect of material flexibility on its ability to self-heal, the influence of the type of metal salt used and the L/M ratio has also been demonstrated [92]. The properties of counterions can affect imidazole/zinc interactions due to the differences in their electronegativity and size. POSS-B was used to study the effect of three zinc(II) salts with counterions [(bis(trifluoromethylsulfonyl)imide—NTf_2_^−^; trifluoromethanesulfonate—OTf^−^; and chloride, Cl^−^) of increasing size (Cl^−^ < OTf^-^ < NTf_2_^−^) and different nature (e.g., electronegativity of Cl^−^ > NTf_2_^−^). The materials prepared with Zn(OTf)_2_ were more rigid than those prepared with Zn(NTf_2_)_2_ at identical L/M ratios. It was due to the higher mobility of the larger NTf_2_^−^ ions, which promoted rapid ligand exchange. Furthermore, the L/M ratio affected the mechanical properties through differences in the crosslinking density. When the L/M ratio was increased from 2 to 6, the materials prepared with ZnCl_2_ exhibited a decrease in glass transition temperature (from 61.9 °C to −21.9 °C), while the highest T_g_ values were observed for composites with Zn(NTf_2_)_2_ and Zn(OTf)_2_ with L/M ratio = 3.5–4.0. Additionally, a significant increase in the Young’s modulus, with a decreasing L/M ratio, was noted for all nanocomposites (e.g., for POSS-B/Zn(NTf_2_)_2_, E = 627 MPa when L/M = 4 and E = 11.2 MPa when L/M = −6). At the same time, an inverse relationship was observed for the ultimate tensile values (e.g., for POSS-B/Zn(NTf_2_)_2_, ε_max_ = 1% when L/M = 4 and ε_max_ = 343% when L/M = 6).

**Figure 28:**
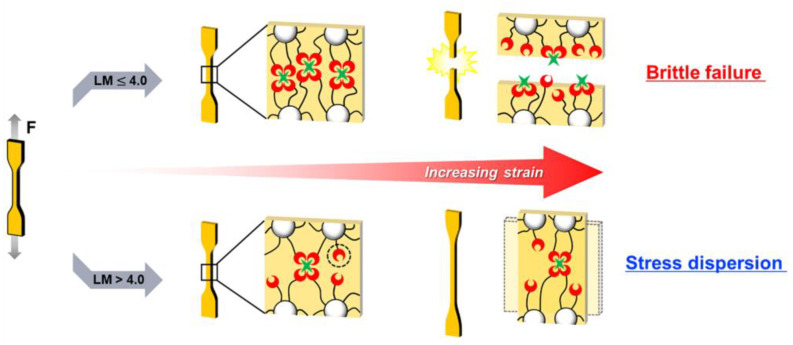
Schematic illustration of the influence of L/M ratio on the mechanical response under tensile tests. Reprinted from *Mater. Today Chem*. 2021, *22*, Sasaki, Y.; Yamamoto, T.; Mori, H., Mechanically robust, ion-conductive, self-healing glassy hybrid materials via tailored Zn/imidazole interaction, 100611, [92]. Copyright (2021), with permission from Elsevier.

**Figure 29:**
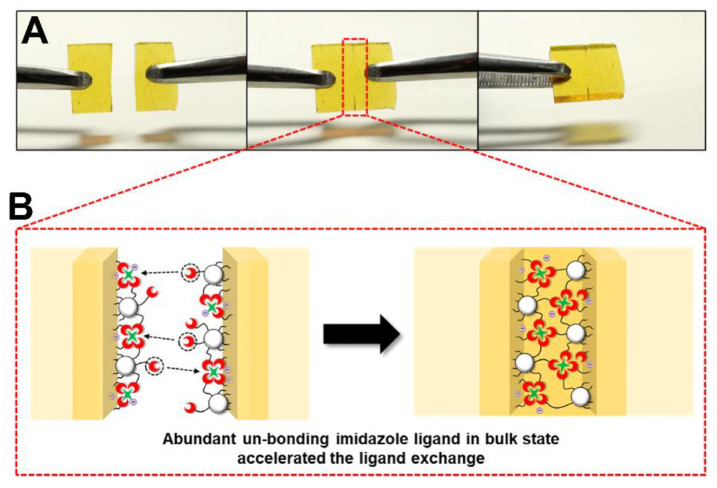
(**A**) Self-healing of POSS-B/Zn(OTf)_2_ sample after 30 s of direct contact between fragments; (**B**) Illustration of self-healing mechanism accelerated by the presence of free imidazole groups. Reprinted from *Mater. Today Chem.* 2021, *22*, Sasaki, Y.; Yamamoto, T.; Mori, H., Mechanically robust, ion-conductive, self-healing glassy hybrid materials via tailored Zn/imidazole interaction, 100611, [92]. Copyright (2021), with permission from Elsevier.

Detailed studies revealed that the ligand exchange rate and L/M ratio in Zn(II)-imidazole complexes also affected the self-healing process. For materials with L/M ≤ 4, all imidazole groups were coordinated to cations, resulting in low ligand exchange dynamics and brittle fracture under deformation (Figure 28). The free imidazole groups in the nanocomposites with L/M > 4 enabled the reduction of local stress concentration by rearrangement of the crosslinking points, resulting in the high strain at fracture. In all the studied cases, the self-healing efficiency reached approximately 90% of the original mechanical strength after rejoining the pieces of cut samples for 30 s at room temperature. The presence of a small excess of imidazole moieties at the cut edge accelerated ligand exchange resulting in good self-healing properties with a slight decrease in mechanical strength after the repair. The effect of the L/M ratio and the presence of free imidazole groups was confirmed by AFM studies of adhesion strength for fractured and surface layers (Figure 29).

## 5. POSS-Containing Self-Healing Hydrogels

Hydrogels with a three-dimensional network structure, consisting of crosslinked natural or synthetic polymers, are widely used in biomedicine and biotechnology due to their good biocompatibility and customizable physicochemical properties [93,94,95,96]. These “soft and wet” materials can retain large amounts of water or biological fluids under physiological conditions. The gelation process under mild conditions can result from physical association (changes in pH, temperature, ionic interactions, host/guest interactions) and/or chemical crosslinking (e.g., Michael addition, Schiff base, or disulfide bond formation). Supramolecular hydrogels based on non-covalent interactions can exhibit interesting physical properties and functions, including self-healing and responsiveness to stimuli. The former is particularly important because lack of elasticity makes hydrogels susceptible to damage or deformation under external mechanical forces. In the case of injectable hydrogels used as tissue scaffolds, such damage can lead to infection and induction of inflammatory responses. However, non-covalent supramolecular networks can exhibit an autonomous self-healing capacity that helps restore their functionality and structure after damage. Additionally, they can be functionalized by modifying host or guest units. Hydrogels prepared from a combination of small host/guest monomers appear to have more favorable complexation properties than those formed from more sterically hindered macromolecular host/guest systems [97,98]. Functionalized polyhedral oligomeric silsesquioxanes, which have a specific 3D structure, may be a good platform for the design and preparation of such materials. Moreover, POSS have been shown to be non-toxic and cytocompatible [99].

**Figure 30:**
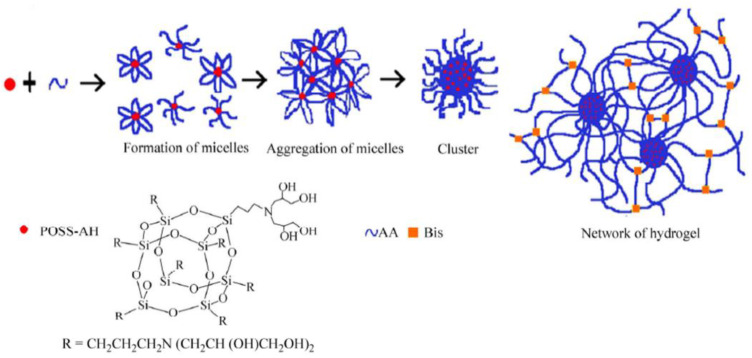
Formation of a dual network in POSS-AH/PAA hydrogels. Reprinted by permission from Springer-Verlag, *Chin. J. Polym. Sci*. 2016, *34*, 185–194. Highly Stretchable and Self-healing Hydrogels Based on Poly(acrylic acid) and Functional POSS. Yang, L.-Q.; Lu, L.; Zhang, C.-W.; Zhou, C.-R.) [100].

Self-regenerating hydrogels with high mechanical strength [stretch above 4200% relative to initial length, relatively high tensile strength (0.1–0.25 MPa), and notch insensitivity] were prepared by in situ crosslinking of self-assembling colloidal poly(acrylic acid) micelles functionalized with POSS [100]. The addition of POSS molecules functionalized with N,N-di(2,3-dihydroxypropyl)-(aminopropyl) groups (POSS-AH) to PAA solution led to the growth of micelles, followed by the formation of a dual crosslinked network (Figure 30). Crosslinks were formed by hydrogen bonds and ionic interactions between PAA chains and POSS-AH, as well as by covalent bonds between PAA and bis(N,N’-methylenebis-acrylamide).

The POSS-AH/PAA hydrogel had the ability to self-heal due to reversible physical crosslinks. The cut samples were stored in a closed vessel at 37 °C for 48 h and sealed almost completely. Tensile tests performed on the original and self-healing samples showed that the POSS-AH content, AA monomer concentration, and AA neutralization degree had significant quantitative effects on the self-healing process. POSS-AH were effectively crosslinking PAA, but an increase in the silsesquioxane concentration led to a decrease in the ability of hydrogels to self-heal. This effect may be due to the fact that as the amount of POSS increases, the lengths of the flexible chains on the cut surface decrease. The results of the tensile test illustrate the effect of POSS-AH on the self-healing process (50% recovery of tensile strength after healing when POSS-AH was applied at 5 wt.% relative to AA and 20% recovery at 10 wt.%). A determining factor in the self-healing process is thus the appropriate amount of long polymer chains that must be present on the cut surfaces [101]. Accordingly, the level of macromolecular entanglement increased and the mechanical strength of the hydrogel was significantly improved. This correlated with the observation that the self-healing ability of the POSS-AH/PAA hydrogel increases as the monomer concentration in the reaction system at the beginning of the reaction (and consequently, the PAA concentration) increases. The enhanced degree of AA neutralization was obtained by increasing the content of NaOH necessary to maintain the pH value of the system appropriate for a physiological environment. This disrupted the hydrogen bonding in PAA and caused a decrease in self-healing ability and mechanical strength

**Figure 31:**
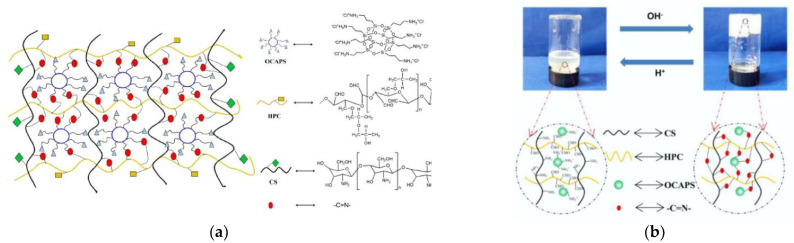
Structure of CS/HPC/POSS hydrogels (**a**) and their pH responsivity (**b**). Reprinted from *React. Funct. Polym.* 2021, *158*, Zhang, X.; Meng, Y.; Shen, W.; Dou, J.; Liu, R.; Jin, Q.; Fang, S., pH-responsive injectable polysaccharide hydrogels with self-healing, enhanced mechanical properties based on POSS, 104773, Ref. [102] Copyright (2021), with permission from Elsevier.

An injectable and self-healing POSS-based hydrogel was also prepared using water-soluble octa(γ-chloroammoniumpropyl)-silsesquioxane (OCAPS), which was mixed with a chitosan (CS) solution and then reacted with the aldehyde groups of oxidized hydroxypropyl cellulose (HPC) [102]. OCAPS acted as a chemical crosslinking agent, using amino groups to form imide bonds with HPC. As a consequence, a non-toxic and biodegradable nanocomposite hydrogel (CS/HPC/POSS) was prepared using Schiff base chemistry (Figure 31). FTIR spectroscopy studies of the hydrogels revealed the presence of absorption peaks of Schiff base bonds (-C=N-) absorption peaks in the range of 1690–1620 cm^−1^ as well as stretching and bending vibrations of N-H bonds (3500–3400 cm^−1^ and 1630–1550 cm^−1^, respectively). The ν(N-H) were sharper in CS/HPC/POSS than those in CS/HPC hydrogels, indicating that hydrogen bonding interactions between CS molecules have been reduced in the hybrid hydrogels. X-ray diffraction studies revealed that the incorporation of OCAPS also affects the ordered arrangement of HPC molecules and makes the hydrogels more amorphous. Cross-sectional SEM images of freeze-dried CS/HPC/POSS showed that the internal structure of the hydrogels consists of pores with thin walls. Energy dispersive X-ray spectroscopy (EDS) showed that OCAPS molecules were well dispersed and uniformly distributed throughout the matrix.

**Figure 32:**
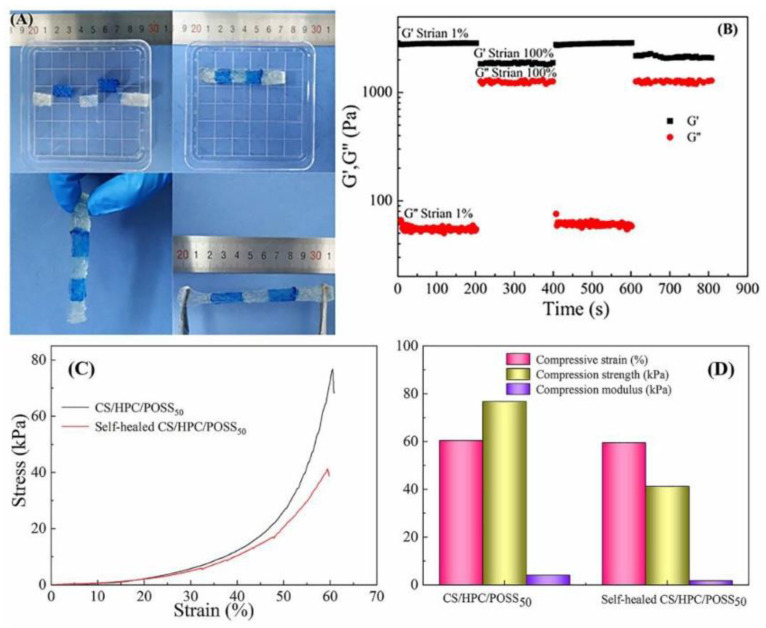
Self-healing performance (**A**) and continuous step strain measurements (**B**) of CS/HPC/POSS_40_ hydrogel; Mechanical properties and results of compression tests on the original and self-healed CS/HPC/POSS_50_ hydrogel (**C**,**D**). Reprinted from *React. Funct. Polym.* 2021, *158*, Zhang, X.; Meng, Y.; Shen, W.; Dou, J.; Liu, R.; Jin, Q.; Fang, S., pH-responsive injectable polysaccharide hydrogels with self-healing, enhanced mechanical properties based on POSS, 104773, Ref. [102] Copyright (2021), with permission from Elsevier.

The readily formed dynamic Schiff bonds were responsible for pH sensitivity and self-healing, while the incorporation of OCAPS improved the mechanical properties of the hydrogel. The gelation time, degree of swelling, degradation, compressive modulus, and strength of CS/HPC/POSS hydrogels were regulated by changing the amount of OCAPS. In general, CS/HPC/POSS hydrogels exhibited a faster swelling than CS/HPC hydrogels. Their equilibrium swelling ratio first increased and then decreased with increasing OCAPS content. The reversible sol-gel transition for CS/HPC/POSS could be controlled by adjusting the pH value. When the pH of the environment was lower than 4.2, the crosslinking density of CS/HPC/POSS was low (Schiff base bond cleavage). When the pH value was increased then the initially liquid CS/HPC/POSS mixture gradually transformed into a solid gel. It was also demonstrated (using bovine serum albumin as a model) that the hydrogel was suitable for drug loading and release. It should be noted that drug release from hybrid hydrogels improved after the introduction of OCAPS. The self-recovery of the hybrid hydrogel was evaluated by continuous step strain measurements according to the strain amplitude results (Figure 32B). The results suggested that the hydrogel network had a good self-repairing ability. The compression strain of the self-healed hydrogel was close to the original value (Figure 32C). However, the compression strength and modulus significantly decreased (Figure 32D).

**Figure 33:**
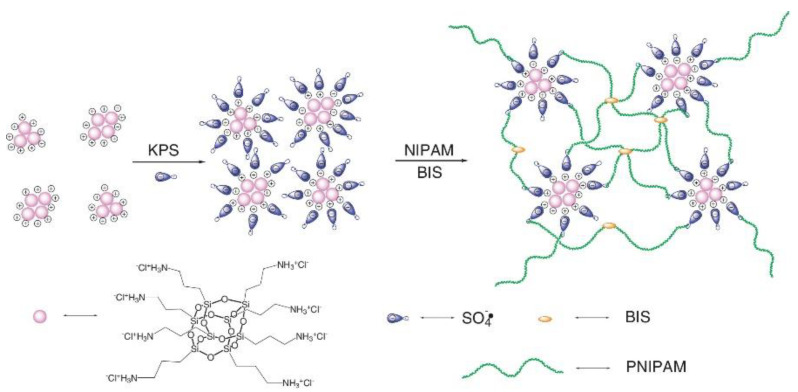
Formation of OCAPS-PNIPAM hydrogels. Copyright (2020) Wiley. Used with permission from Zhang, X.; Shen, W.; Dou, J.; Meng, Y.; Fang, S.; Liu, R., Enhanced mechanical properties and self-healing behavior of PNIPAM nanocomposite hydrogel by using POSS as a physical crosslinker. *J. Appl. Polym. Sci.* 2020, *137*, Article number 48486. WILEY-VCH Verlag GmbH & Co. KGaA, Weinheim [103].

Due to electrostatic interactions and the formation of hydrogen bonds between OCAPs and amide linkers in poly(N-isopropylacrylamide) (PNIPAM) chains, silsesquioxane molecules could also act as effective physical crosslinkers in a nanocomposite hydrogel (OCAPS-PNIPAM), which significantly reduced the loading of N,N′-methylenebisacrylamide (BIS) (50–100 ppm) used as a chemical crosslinking agent [103]. Water-soluble OCAPS molecules aggregated in solution into nanoparticles with 7–20 nm size and positive charge (127.6–213.7 mV), which attracted persulfate anion radical that initiated in situ polymerization of N-isopropylacrylamide (Figure 33). The incorporation of OCAPS reduces the swelling ratio of PNIPAM hydrogel in deionized water and leads to an enhancement of mechanical properties of OCAPS-PNIPAM hydrogel (compression modulus: 3.52–7.59 kPa, elasticity modulus 7.67–33.91 kPa, tensile strength 6.82–243.41 kPa, fracture energy 503.5–4781.7 J·m^−2^) that can be adjusted by the feed ratio.

OCAPS-PNIPAM hydrogels of low loading of the hybrid silsesquioxane crosslinker (<< 7%) can self-heal under certain conditions. This suggests that at very low concentration of BIS, chemical crosslinking of PNIPAM is limited and occurs in microscopic domains. Additionally, the higher OCAPS content limits interactions between PNIPAM macromolecules, which is essential for efficient healing. The 3% OCAPS-PNIPAM0.005 hydrogel, which exhibited self-repairing properties, was subjected to a continuous step-strain sweep test. It was found that the material self-healed as a result of periodic changes in continuous step strain, consistent with the process of damage and reconstruction of the physically crosslinked network.

Viscoelasticity and smart bidirectional shape-memory behavior of hydrogels formed from N-isopropylacrylamide (NIPAm) and (propyl methacrylate)heptaisobutyl)-POSS copolymers were also investigated [104]. PNIPAm-POSS copolymers are untangled and exhibit a thermoplastic and thermoresponsive hydrogel behavior, with covalently linked POSS forming physical crosslinks located in separated nanodomains. This results in the elastic behavior of unentangled copolymer melts. The hydrogels self-healed and exhibited liquid-like properties under high amplitude shear and rubber-like behavior immediately after shear cessation. This indicated that POSS aggregates can act as dynamic crosslinkers that dissociate and re-form during shear. The physical hydrogels of the honeycomb structure exhibited significant swelling, greater than the chemically crosslinked hydrogels with the same POSS content, that were obtained by reacting the NIPAm and POSS monomers with 0.13 mM of *N*,*N*′-methylenebisacrylamide. Morphological studies showed that the pore size and swelling of these materials were a decreasing function of POSS content. A thermally activated and reversible shape memory behavior was observed for PNIPAm-POSS, which involved swelling-deswelling and can be used for drug delivery.

**Figure 34:**
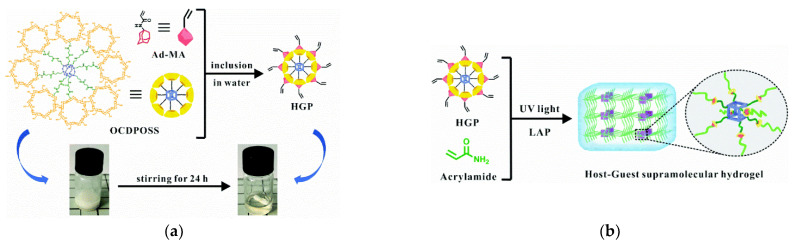
Structure of a POSS-based supramolecular crosslinker (**a**) and formation of a hydrogel based on reversible host–guest interactions (**b**). Reproduced from *Biomater. Sci.* 2020, *8*, 3359–3369 [105], with permission from the Royal Society of Chemistry.

**Figure 35:**
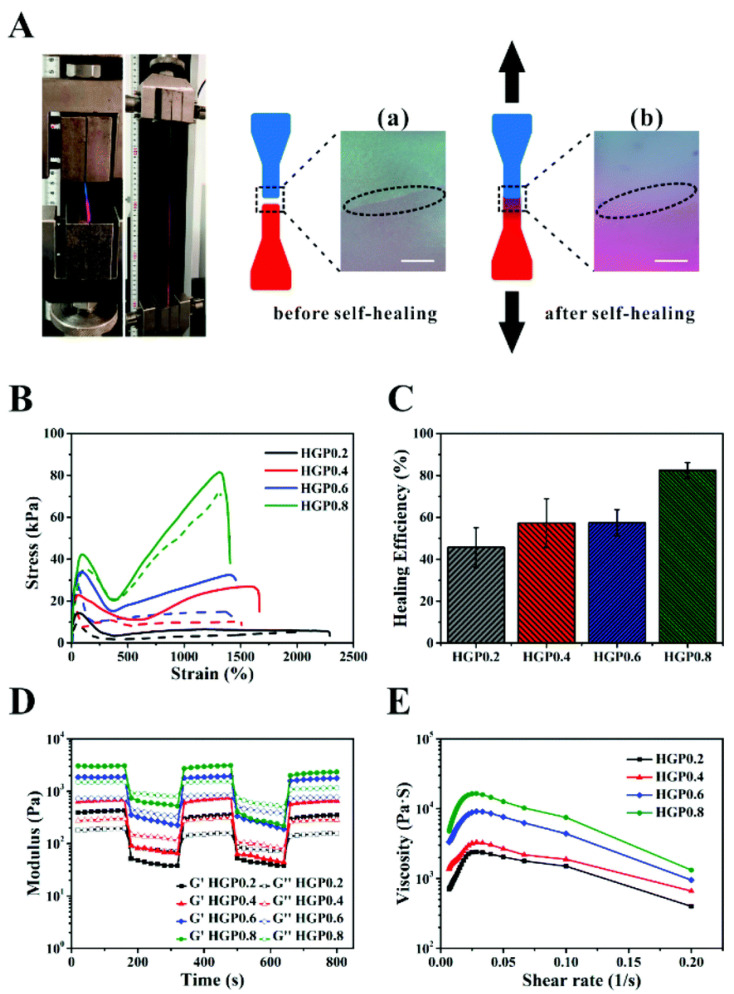
Self-healing and shear thinning properties of HGP hydrogels: (**A**) optical microscopy images of samples before and after self-healing (scale bar: 500 μm); (**B**) stress–strain curves of the HGP hydrogels (― original and −−− self-healed) with different content of POSS; (**C**) the self-healing efficiency based on rupture energy; (**D**) amplitude oscillatory sweep (1% strain–500% strain– 1% strain); (**E**) shear stress of the HGP hydrogels loading a shear rate sweep. Reproduced from *Biomater. Sci.* 2020, *8*, 3359–3369 [105] with permission from the Royal Society of Chemistry.

Another approach to a POSS-hydrogel hybrid system was based on the formation of supramolecular complexes through structural relationships and non-covalent binding [105]. Supramolecular interactions between octa-(cyclodextrin-functionalized) polyhedral silsesquioxane (OCDPOSS) and acrylamide-modified adamantane (Ad-AAm) resulted in the formation of a star-shaped POSS crosslinker (HGP). Molecules of the supramolecular crosslinking agent were copolymerized with acrylamide by a photoinitiated process (Figure 34). The microstructure of the host-guest POSS-hydrogel network was examined with SEM. The resulting injectable hydrogels exhibited super-stretchability and self-healing properties as demonstrated by tensile and rheological measurements. These supramolecular hybrid materials also underwent rapid self-healing and shear-thinning due to the dynamic host-guest interactions with reversible cleavage and reconstitution. Their mechanical strength may be related to the presence of rigid POSS as the core of the supramolecular crosslinker, while the multivalent host-guest interactions improve the ductility, injectability, and overall efficiency of the self-healing process (Figure 35). The stress-strain curves of the self-healed samples and the original hydrogels were similar. The self-repaired material retained good ductility with high strain. As the crosslinking concentration of HGP increased from 0.2 to 0.8 mol%, the self-healing efficiency was improved (from 46% to 81%). The results of the amplitude oscillatory sweep tests with the alternating strains of 1% and 500% provided insight into the dynamic properties of HGP hydrogels. At high strain, the materials exhibited liquid-like behavior (G″ > G′) due to damage of the internal hydrogel structures. However, the value of G′ immediately increased up to the initial modulus value, suggesting that although the HGP hydrogels were broken under high strain, they were capable of self-healing in a very short time. The shear-thinning behavior of hydrogels under high shear rates was also evidenced. Rheological measurements showed that the reversed host-guest interactions can reform rapidly with decreasing shear rates, which is promising for the preservation of the hydrogel network after injection into tissue. The biocompatible supramolecular hydrogels are suitable for sustained drug release because the hydrophobic voids of cyclodextrin units can be used as carriers for hydrophobic small molecules.

**Figure 36:**
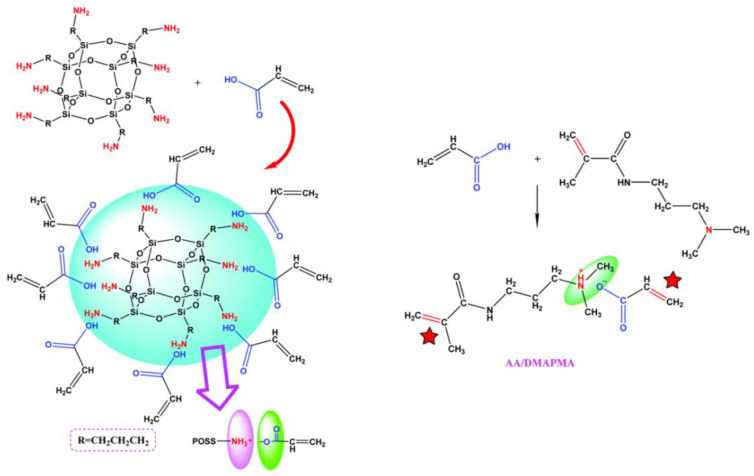
Synthesis of a hydrogel based on POSS–NH_2_–AA. Reproduced from *Soft Matter* 2017, *13*, 5645–5648 [106] with permission from the Royal Society of Chemistry.

A highly stretchable and self-healing ampholytic hydrogel was prepared using octa(aminopropyl)silsesquioxane (POSS-NH_2_) and acrylic acid (Figure 36) [106]. The resulting clear solution (POSS–NH_2_–AA) can be mixed with other monomers [e.g., *N*-[3-(dimethylamino)propyl]methacrylamide (DMAPMA)] to obtain molecularly dispersed systems. The presence of POSS, acting as an amphoteric and noncovalent macro-crosslinker in radical copolymerization, reinforced the hydrogel structure. The synthetic hydrogel is sensitive to pH changes and exhibits a rapid self-healing ability (stress of 8 kPa) and excellent tensile mechanical properties (strain of 4683% and stress of 37.8 kPa). The swelling index of the material under acidic and alkaline conditions was higher than that under neutral conditions. The hydrogel also showed good cohesion with plastics, glass, and metal. The inorganic core of POSS units molecularly dispersed in the hydrogel matrix strengthened it by enhancing the intermolecular forces. Moreover, the electrostatic interactions in these hydrogels, favorably influenced the self-healing properties.

## 6. Conclusions

The review covers the literature reports on POSS-containing self-healing materials published within the last 14 years. The number of papers devoted to this topic is growing which proves a significant potential of POSS in this area. Such self-healing systems connect nanotechnology and smart materials and despite being, at the moment, a relatively small niche, compared to the large number of papers devoted to polymeric-self healing materials, the specific properties of polyhedral silsesquioxanes may present a unique opportunity for next-generation self-healing and dynamic systems, including materials designed for biomedicine and biotechnology. SHM containing polyhedral oligomeric silsesquioxanes as building blocks can have unique properties. Their design should take into account both the chemical structure of POSS derivatives and the specific application area for a given SHM. Various factors may influence the self-repairing activity of POSS-based polymer nanocomposites, hydrophobic coatings, or hydrogels. Appropriate functionalization enables, for example, the formation of dynamic bonds with POSS molecules as reactive components or can influence the intrinsic properties of SHM (e.g., fluorinated POSS for superhydrophobic and oleophobic coatings). The macromolecular dynamics required for effective self-healing can be achieved by reversibly releasing bulky POSS cages through DA reactions, metal-ligand interactions, or thiol/disulfide chemistry. Another approach is the formation of separated inclusions in the polymer matrix through hydrophobic interactions and POSS aggregation. The specific chemistry of some of the interactions occurring in POSS-based SHMs makes them interesting next-generation materials with improved mechanical properties, suitable for applications in sensors, nanostructured superhydrophobic coatings, and objects with shape recovery properties.

## Data Availability

Not applicable.

## References

[1] van der Zwaag S. (2007). Self Healing Materials: An Alternative Approach to 20 Centuries of Materials Science.

[2] Zhang W., Jiang H., Chang Z., Wu W., Wu G., Wu R., Li J. (2020). Recent achievements in self-healing materials based on ionic liquids: A review. J. Mater. Sci..

[3] Rahman M.W., Shefa N.R. (2021). Minireview on Self-Healing Polymers: Versatility, Application, and Prospects. Adv. Polym. Techn..

[4] Ekeocha J., Ellingford C., Pan M., Wemyss A.M., Bowen C., Wan C. (2021). Challenges and Opportunities of Self-Healing Polymers and Devices for Extreme and Hostile Environments. Adv. Mater..

[5] Zhang K., Sun J., Song J., Gao C., Wang Z., Song C., Wu Y., Liu Y. (2020). Self-Healing Ti_3_C_2_ MXene/PDMS Supramolecular Elastomers Based on Small Biomolecules Modification for Wearable Sensors. ACS Appl. Mater. Interf..

[6] Tepper R., Bode S., Geitner R., Jäger M., Görls H., Vitz J., Dietzek B., Schmitt M., Popp J., Hager M.D. (2017). Polymeric Halogen-Bond-Based Donor Systems Showing Self-Healing Behavior in Thin Films. Angew. Chem.-Int. Ed..

[7] Chen X., Dam M.A., Ono K., Mal A., Shen H., Nutt S.R., Sheran K., Wudl F. (2002). A Thermally re-mendable cross-linked polymeric material. Science.

[8] Kavitha A.A., Singha N.K. (2009). “Click chemistry” in tailor-made polymethacrylates bearing reactive furfuryl functionality: A new class of self-healing polymeric material. ACS Appl. Mater. Interfaces.

[9] Mondal P., Behera P.K., Singha N.K. (2017). A healable thermo-reversible functional polymer prepared via RAFT polymerization and ultrafast ‘click’ chemistry using a triazolinedione derivative. Chem. Commun..

[10] Fawcett A.S., Brook M.A. (2014). Thermoplastic Silicone Elastomers through Self-Association of Pendant Coumarin Groups. Macromolecules.

[11] Jin J., Cai L., Jia Y.-G., Liu S., Chen Y., Ren L. (2019). Progress in self-healing hydrogels assembled by host–guest interactions: Preparation and biomedical applications. J. Mater. Chem. B.

[12] Rambarran T., Bertrand A., Gonzaga F., Boisson F., Bernard J., Fleury E., Ganachaud F., Brook M.A. (2016). Sweet supramolecular elastomers from α,ω-(β-cyclodextrin terminated) PDMS. Chem. Commun..

[13] Ming X., Du J., Zhang C., Zhou M., Cheng G., Zhu H., Zhang Q., Zhu S. (2021). All-Solid-State Self-Healing Ionic Conductors Enabled by Ion–Dipole Interactions within Fluorinated Poly(Ionic Liquid) Copolymers. ACS Appl. Mater. Interfaces.

[14] Voorhaar L., Diaz M.M., Leroux F., Rogers S., Abakumov A.M., Van Tendeloo G., Van Assche G., Van Mele B., Hoogenboom R. (2017). Supramolecular thermoplastics and thermoplastic elastomer materials with self-healing ability based on oligomeric charged triblock copolymers. NPG Asia Mater..

[15] Li C.-H., Zuo J.-L. (2020). Self-Healing Polymers Based on Coordination Bonds. Adv. Mater..

[16] Mozhdehi D., Ayala S., Cromwell O.R., Guan Z. (2014). Self-Healing Multiphase Polymers via Dynamic Metal−Ligand Interactions. J. Am. Chem. Soc..

[17] Tuncaboylu D.C., Sari M., Oppermann W., Okay O. (2011). Tough and Self-Healing Hydrogels Formed via Hydrophobic Interactions. Macromolecules.

[18] Liu Y.-L., Chuo T.-W. (2013). Self-healing polymers based on thermally reversible Diels–Alder chemistry. Polym. Chem..

[19] Nicolaou K.C., Snyder S.A., Montagnon T., Vassilikogiannakis G. (2002). The Diels-Alder reaction in total synthesis. Angew. Chem. Int. Ed..

[20] Kavitha A.A., Singha N.K. (2010). Smart “all acrylate” ABA triblock copolymer bearing reactive functionality via atom transfer radical polymerization (ATRP): Demonstration of a “click reaction” in thermoreversible property. Macromolecules.

[21] Pramanik N.B., Nando G.B., Singha N.K. (2015). Self-healing polymeric gel via RAFT polymerization and Diels–Alder click chemistry. Polymer.

[22] Nasresfahani A., Zelisko P.M. (2017). Synthesis of a self-healing siloxane-based elastomer cross-linked via a furan-modified polyhedral oligomeric silsesquioxane: Investigation of a thermally reversible silicon-based cross-link. Polym. Chem..

[23] De Bruycker K., Billiet S., Houck H.A., Chattopadhyay S., Winne J.M., Du Prez F.E. (2016). Triazolinediones as highly enabling synthetic tools. Chem. Rev..

[24] Banerjee S.L., Bhattacharya K., Samanta S., Singha N.K. (2018). Self-healable antifouling zwitterionic hydrogel based on synergistic phototriggered dynamic disulfide metathesis reaction and ionic interaction. ACS Appl. Mater. Interfaces.

[25] Lv C., Zhao K., Zheng J. (2018). A Highly Stretchable Self-Healing Poly(dimethylsiloxane) Elastomer with Reprocessability and Degradability. Macromol. Rapid Commun..

[26] Michal B.T., Spencer E.J., Rowan S.J. (2016). Stimuli-responsive reversible two-level adhesion from a structurally dynamic shape-memory polymer. ACS Appl. Mater. Interf..

[27] Michal B.T., Jaye C.A., Spencer E.J., Rowan S.J. (2013). Inherently Photohealable and Thermal Shape-Memory Polydisulfide Networks. ACS Macro Lett..

[28] Kathan M., Kovaricek P., Jurissek C., Senf A., Dallmann A., Thuenemann A.F., Hecht S. (2016). Control of Imine Exchange Kinetics with Photoswitches to Modulate Self-Healing in Polysiloxane Networks by Light Illumination. Ang. Chem. Inter. Ed..

[29] Li C.-H., Wang C., Keplinger C., Zuo J.-L., Jin L., Sun Y., Zheng P., Cao Y., Lissel F., Linder C. (2016). A highly stretchable autonomous self-healing elastomer. Nat. Chem..

[30] Zhang H., Cai C., Liu W., Li D., Zhang J., Zhao N., Xu J. (2017). Recyclable Polydimethylsiloxane Network Crosslinked by Dynamic Transesterification Reaction. Sci. Rep..

[31] Lai J.-C., Mei J.-F., Jia X.-Y., Li C.-H., You X.-Z., Bao Z. (2016). A Stiff and Healable Polymer Based on Dynamic-Covalent Boroxine Bonds. Adv. Mater..

[32] Hua J., Liu C., Fei B., Liu Z. (2022). Self-Healable and Super-Tough Double-Network Hydrogel Fibers from Dynamic Acylhydrazone Bonding and Supramolecular Interactions. Gels.

[33] Nevejans S., Ballard N., Miranda J.I., Reck B., Asua J.M. (2016). The underlying mechanisms for self-healing of poly(disulfide)s. Phys. Chem. Chem. Phys..

[34] Li J., Huang H., Fielden M., Pan J., Ecco L., Schellbach C., Delmas G., Claesson P.M. (2016). Towards the mechanism of electrochemical activity and self-healing of 1 wt% PTSA doped polyaniline in alkyd composite polymer coating: Combined AFM-based studies. RSC Adv..

[35] Sanka R.V.S.P., Krishnakumar B., Leterrier Y., Pandey S., Rana S., Michaud V. (2019). Soft Self-Healing Nanocomposites. Front. Mater..

[36] Cordes D.B., Lickiss P.D., Rataboul F. (2010). Recent Developments in the Chemistry of Cubic Polyhedral Oligosilsesquioxanes. Chem. Rev..

[37] Li Y., Dong X.-H., Zou Y., Wang Z., Yue K., Huang M., Liu H., Feng X., Lin Z., Zhang W. (2017). Polyhedral oligomeric silsesquioxane meets “click” chemistry: Rational design and facile preparation of functional hybrid materials. Polymer.

[38] Ullah A., Ullah S., Khan G.S., Shah S.M., Hussain Z., Muhammad S., Siddiq M., Hussain H. (2016). Water soluble polyhedral oligomeric silsesquioxane based amphiphilic hybrid polymers: Synthesis, self-assembly, and applications. Eur. Polym. J..

[39] Kowalewska A. (2017). Self-assembling polyhedral silsesquioxanes-Structure and properties. Curr. Org. Chem..

[40] Kowalewska A., Kalia S., Pielichowski K. (2018). Self-assembly of POSS-Containing Materials. Polymer/POSS Nanocomposites and Hybrid Materials, Preparation, Properties, Applications.

[41] Chi H., Wang M., Xiao Y., Wang F., Joshy K.S. (2018). Self-Assembly and Applications of Amphiphilic Hybrid POSS Copolymers. Molecules.

[42] Chen F., Lin F., Zhang Q., Cai R., Wu Y., Ma X. (2019). Polyhedral Oligomeric Silsesquioxane Hybrid Polymers: Well-Defined Architectural Design and Potential Functional Applications. Macromol. Rapid Commun..

[43] Joshi M., Butola B.S. (2004). Polymeric nanocomposites-Polyhedral oligomeric silsesquioxanes (POSS) as hybrid nanofiller. J. Macromol. Sci.-Polym. Rev..

[44] Kuo S.-W., Chang F.-C. (2011). POSS related polymer nanocomposites. Progr. Polym. Sci..

[45] Zhang W., Camino G., Yang R. (2017). Polymer/polyhedral oligomeric silsesquioxane (POSS) nanocomposites: An overview of fire retardance. Progr. Polym. Sci..

[46] Shi H., Yang J., You M., Li Z., He C. (2020). Polyhedral Oligomeric Silsesquioxanes (POSS)-Based Hybrid Soft Gels: Molecular Design, Material Advantages, and Emerging Application. ACS Mater. Lett..

[47] Dong F., Lu L., Ha C.-S. (2019). Silsesquioxane-Containing Hybrid Nanomaterials: Fascinating Platforms for Advanced Applications. Macromol. Chem. Phys..

[48] Liu S., Guo R., Li C., Lu C., Yang G., Wang F., Nie J., Ma C., Gao M. (2021). POSS hybrid hydrogels: A brief review of synthesis, properties and applications. Eur. Polym. J..

[49] Wang C., Zhou L., Du Q., Shan T., Zheng K., He J., He H., Chen S., Wang X. (2022). Synthesis, properties and applications of well-designed hybrid polymers based on polyhedral oligomeric silsesquioxane. Polym. Int..

[50] Xu S., Zhao B., Adeel M., Zheng S. (2019). Shape Memory and Self-Healing Properties of Poly(acrylate amide) Elastomers Reinforced with Polyhedral Oligomeric Silsesquioxanes. ACS Appl. Polym. Mater..

[51] Zhao B., Xu S., Zheng S. (2019). Synthesis, self-assembly and self-healing properties of organic-inorganic ABA triblock copolymers with poly(POSS acrylate) endblocks. Polym. Chem..

[52] Strachota B., Matějka L., Hodan J., Kobera L., Mahun A., Dybal J., Šlouf M. (2021). Polyhedral oligomeric silsesquioxane (POSS)-based epoxy nanocomposite involving a reversible Diels–Alder-type network as a self-healing material. J. Adhes. Sci. Technol..

[53] Strąkowska A., Kosmalska A., Masłowski M., Szmechtyk T., Strzelec K., Zaborski M. (2019). POSS as promoters of self-healing process in silicone composites. Polym. Bull..

[54] Guadagno L., Naddeo C., Raimondo M., Barra G., Vertuccio L., Sorrentino A., Binder W.H., Kadlec M. (2017). Development of self-healing multifunctional materials. Compos. Part B.

[55] Jing Z., Shi X., Zhang G., Gu J. (2017). Synthesis and properties of poly(lactide)/poly(ε-caprolactone) multiblock supramolecular polymers bonded by the self-complementary quadruple hydrogen bonding. Polymer.

[56] Zhou B., He D., Hu J., Ye Y., Peng H., Zhou X., Xie X., Xue Z. (2018). A Flexible, Self-healing and Highly Stretchable Polymer Electrolyte via Quadruple Hydrogen Bonding for Lithium-ion Batteries. J. Mater. Chem. A.

[57] Yan X., Liu Z., Zhang Q., Lopez J., Wang H., Wu H.-C., Niu S., Yan H., Wang S., Lei T. (2018). Quadruple H-Bonding Cross-Linked Supramolecular Polymeric Materials as Substrates for Stretchable, Antitearing, and Self-healable Thin Film Electrodes. J. Am. Chem. Soc..

[58] Song Y., Liu Y., Qi T., Li G.L. (2018). Towards Dynamic but Supertough Healable Polymers through Biomimetic Hierarchical Hydrogen-Bonding Interactions. Angew. Chem. Int. Ed..

[59] Hentschel J., Kushner A.M., Ziller J., Guan Z. (2012). Self-healing Supramolecular Block Copolymers. Angew. Chem. Int. Ed..

[60] Yari H., Mohseni M., Messori M. (2016). A scratch resistant yet healable automotive clearcoat containing hyperbranched polymer and POSS nanostructures. RSC Adv..

[61] Xiang S., Liu W. (2021). Self-healing superhydrophobic surfaces: Healing principles and applications. Adv. Mater. Interfaces.

[62] Wang H., Xue Y., Ding J., Feng L., Wang X., Lin T. (2011). Durable, Self-healing superhydrophobic and superoleophobic surfaces from fluorinated-decyl polyhedral oligomeric silsesquioxane and hydrolyzed fluorinated alkyl silane. Angew. Chem. Int. Ed..

[63] Wang H., Zhou H., Gestos A., Fang J., Niu H., Ding J., Lin T. (2013). Robust, electro-conductive, self-healing superamphiphobic fabric prepared by one-step vapour-phase polymerisation of poly(3,4-ethylenedioxythiophene) in the presence of fluorinated decyl polyhedral oligomeric silsesquioxane and fluorinated alkyl silane. Soft Matter.

[64] Wang H., Zhou H., Gestos A., Fang J., Lin T. (2013). Robust, superamphiphobic fabric with multiple self-healing ability against both physical and chemical damages. ACS Appl. Mater. Interfaces.

[65] Zhao R., Chen Y., Liu G., Jiang Y., Chen K. (2018). Fabrication of self-healing waterbased superhydrophobic coatings from POSS modified silica nanoparticles. Mater. Lett..

[66] Golovin K., Boban M., Mabry J.M., Tuteja A. (2017). Designing self-healing superhydrophobic surfaces with exceptional mechanical durability. ACS Appl. Mater. Interfaces.

[67] Yari H., Mohseni M., Messori M., Ranjbar Z. (2014). Tribological properties and scratch healing of a typical automotive nano clearcoat modified by a polyhedral oligomeric silsesquioxane compound. Eur. Polym. J..

[68] Chen K., Zhou J., Ge F., Zhao R., Wang C. (2019). Smart UV-curable fabric coatings with self-healing ability for durable self-cleaning and intelligent oil/water separation. Colloids Surf. A Physicochem. Eng. Asp..

[69] Li H., Miao S., Chen W., Yang X., Li M., Xing T., Zhao Y., Chen G. (2021). Durable superhydrophobic and oleophobic cotton fabric based on the grafting of fluorinated POSS through silane coupling and thiol-ene click reaction. Colloids Surf. A Physicochem. Eng. Asp..

[70] Zhao B., Mei H., Liu N., Zheng S. (2020). Organic−Inorganic Polycyclooctadienes with Double-Decker Silsesquioxanes in the Main Chains: Synthesis, Self-Healing, and Shape Memory Properties Regulated with Quadruple Hydrogen Bonds. Macromolecules.

[71] Zhao B., Ding H., Xu S., Zheng S. (2019). Organic-Inorganic Linear Segmented Polyurethanes Simultaneously Having Shape Recovery and Self-Healing Properties. ACS Appl. Polym. Mater..

[72] Xu S., Zhao B., Raza M., Li L., Wang H., Zheng S. (2020). Shape Memory and Self-Healing Nanocomposites with POSS-POSS Interactions and Quadruple Hydrogen Bonds. ACS Appl. Polym. Mater..

[73] Zhao B., Mei H., Wang H., Li L., Zheng S. (2022). Organic–Inorganic Polyureas with POSS Cages in the Main Chains via Polycondensation of Diamines with Carbon Dioxide. ACS Appl. Polym. Mater..

[74] Behera P.K., Mondal P., Singha N.K. (2018). Self-Healable and Ultrahydrophobic Polyurethane-POSS Hybrids by Diels-Alder "click" Reaction: A New Class of Coating Material. Macromolecules.

[75] Ponnupandian S., Mondal P., Becker T., Hoogenboom R., Lowe A.B., Singha N.K. (2021). Self-healing hydrophobic POSS-functionalized fluorinated copolymers via RAFT polymerization and dynamic Diels–Alder reaction. Polym. Chem..

[76] Raut S.K., Mondal P., Parameswaran B., Sarkar S., Dey P., Gilbert R., Bhadra S., Naskar K., Nair S., Singha N.K. (2021). Self-healable ultrahydrophobic modified bio-based elastomer using Diels-Alder ‘click chemistry’. Eur. Polym. J..

[77] Qi D., Zhang K., Tian G., Jiang B., Huang Y. (2021). Stretchable Electronics Based on PDMS Substrates. Adv. Mater..

[78] Yi B., Wang S., Hou C., Huang X., Cui J., Yao X. (2021). Dynamic siloxane materials: From molecular engineering to emerging applications. Chem. Eng. J..

[79] Huang Y., Yan J., Wang D., Feng S., Zhou C. (2021). Construction of Self-Healing Disulfide-Linked Silicone Elastomers by Thiol Oxidation Coupling Reaction. Polymers.

[80] Cheng C.-C., Chang F.-C., Dai S.A., Lin Y.-L., Lee D.-J. (2015). Bio-complementary supramolecular polymers with effective self-healing functionality. RSC Adv..

[81] Xu Z., Zhao Y., Wang X., Lin T. (2013). A thermally healable polyhedral oligomeric silsesquioxane (POSS) nanocomposite based on Diels-Alder chemistry. Chem. Commun..

[82] Goodman S.H. (1998). Handbook of Thermoset Plastics.

[83] Tian Q., Yuan Y.C., Rong M.Z., Zhang M.Q. (2009). A thermally remendable epoxy resin. J. Mater. Chem..

[84] Chuo T.-W., Liu Y.-L. (2015). Preparation of self-healing organic-inorganic nanocomposites with the reactions between methacrylated polyhedral oligomeric silsesquioxanes and furfurylamine. Compos. Sci. Technol..

[85] Lin H.-K., Liu Y.-L. (2017). Reactive Hybrid of Polyhedral Oligomeric Silsesquioxane (POSS) and Sulfur as a Building Block for Self-Healing Materials. Macromol. Rapid Commun..

[86] Matxain J.M., Asuab J.M., Ruiperez F. (2016). Design of new disulfide-based organic compounds for the improvement of self-healing materials. Phys. Chem. Chem. Phys..

[87] Whittell G.R., Hager M.D., Schubert U.S., Manners I. (2011). Functional soft materials from metallopolymers and metallosupramolecular polymers. Nature Mater..

[88] Bode S., Zedler L., Schacher F.H., Dietzek B., Schmitt M., Popp J., Hager M.D., Schubert U.S. (2013). Self-Healing Polymer Coatings Based on Crosslinked Metallosupramolecular Copolymers. Adv. Mater..

[89] Burnworth M., Tang L., Kumpfer J.R., Duncan A.J., Beyer F.L., Fiore G.L., Rowan S.J., Weder C. (2011). Optically healable supramolecular polymers. Nature.

[90] Sasaki Y., Mori H. (2020). Self-healing hybrids fabricated by metal complexation with imidazole-containing silsesquioxane nanoparticles. Mater. Chem. Front..

[91] Enke M., Bode S., Vitz J., Schacher F.H., Harrington M.J., Hager M.D., Schubert U.S. (2015). Self-healing response in supramolecular polymers based on reversible zinc–histidine interactions. Polymer.

[92] Sasaki Y., Yamamoto T., Mori H. (2021). Mechanically robust, ion-conductive, self-healing glassy hybrid materials via tailored Zn/imidazole interaction. Mater. Today Chem..

[93] Wang W., Narain R., Zeng H. (2018). Rational design of self-healing tough hydrogels: A mini review. Front. Chem..

[94] Ahmed E.M. (2015). Hydrogel: Preparation, characterization, and applications: A review. J. Adv. Res..

[95] Du X., Zhou J., Shi J., Xu B. (2015). Supramolecular Hydrogelators and Hydrogels: From Soft Matter to Molecular Biomaterials. Chem. Rev..

[96] Guo Y., Bae J., Fang Z., Li P., Zhao F., Yu G. (2020). Hydrogels and Hydrogel-Derived Materials for Energy and Water Sustainability. Chem. Rev..

[97] Seiffert S., Sprakel J. (2012). Physical chemistry of supramolecular polymer networks. Chem. Soc. Rev..

[98] Yang B., Wei Z., Chen X., Wei K., Bian L. (2019). Manipulating the mechanical properties of biomimetic hydrogels with multivalent host–guest interactions. J. Mater. Chem. B.

[99] Janaszewska A., Gradzinska K., Marcinkowska M., Klajnert-Maculewicz B., Stanczyk W.A. (2015). In vitro studies of polyhedral oligo silsesquioxanes: Evidence for their low cytotoxicity. Materials.

[100] Yang L.-Q., Lu L., Zhang C.-W., Zhou C.-R. (2016). Highly Stretchable and Self-healing Hydrogels Based on Poly(acrylic acid) and Functional POSS. Chin. J. Polym. Sci..

[101] Zhang H., Xia H., Zhao Y. (2012). Poly(vinyl alcohol) Hydrogel Can Autonomously Self-Heal. ACS Macro Lett..

[102] Zhang X., Meng Y., Shen W., Dou J., Liu R., Jin Q., Fang S. (2021). pH-responsive injectable polysaccharide hydrogels with self-healing, enhanced mechanical properties based on POSS. React. Funct. Polym..

[103] Zhang X., Shen W., Dou J., Meng Y., Fang S., Liu R. (2020). Enhanced mechanical properties and self-healing behavior of PNIPAM nanocomposite hydrogel by using POSS as a physical crosslinker. J. Appl. Polym. Sci..

[104] Romo-Uribe A., Albanil L. (2019). POSS-Induced Dynamic Cross-Links Produced Self-Healing and Shape Memory Physical Hydrogels When Copolymerized with N-Isopropyl acrylamide. ACS Appl. Mater. Interf..

[105] Zhou Y., Zhang Y., Dai Z., Jiang F., Tian J., Zhang W. (2020). A super-stretchable, self-healing and injectable supramolecular hydrogel constructed by a host–guest crosslinker. Biomater. Sci..

[106] Pu W., Jiang F., Chen P., Wei B. (2017). A POSS based hydrogel with mechanical robustness, cohesiveness and a rapid self-healing ability by electrostatic interaction. Soft Matter.

